# CircFAM65A/CANX Condensates Sequester FAM134B to Inhibit ER‐phagy and Suppress Hepatocellular Carcinoma Progression

**DOI:** 10.1002/advs.78469

**Published:** 2026-10-27

**Authors:** Wenxiu Ru, Lunbiao Gan, Yu Lu, Yujing Guo, Qian Huang, Sha Yin, Fengze Nie, Sijia Di, Jiaqi Ning, Siyu Yao, Huanhuan Wan, Le Liu, Zehua Liu, Fa Yang, Weijun Qin, Weihong Wen

**Affiliations:** ^1^ School of Life Sciences and Technology Northwestern Polytechnical University Xi'an China; ^2^ Department of Urology Xijing Hospital Air Force Medical University Xi'an China

**Keywords:** CANX, circFAM65A, condensates, ER‐phagy, hepatocellular carcinoma (HCC), nanomedicine

## Abstract

ER‐phagy has been implicated in tumor progression; however, its regulatory mechanisms in hepatocellular carcinoma (HCC) remain largely unexplored. Here, we identified circFAM65A as a novel circRNA that is downregulated in HCC and its overexpression suppressed HCC progression. Mechanistically, circFAM65A interacted with CANX and attenuated the CANX‐mediated promotion of ER‐phagy and HCC progression. Specifically, circFAM65A promoted CANX condensate formation, and these condensates sequestered FAM134B, reducing the FAM134B‐LC3 interaction and suppressing ER‐phagy. Furthermore, a biomimetic membrane‐coated nanoparticle (PLGA/circFAM65A@M‐3E11) was designed to deliver circFAM65A, which effectively inhibited HCC growth in vivo. Together, these findings identify circFAM65A as a regulator of CANX‐mediated ER‐phagy and support its delivery as a potential therapeutic strategy for HCC.

## Graphical abstract

The novel circRNA circFAM65A suppresses HCC progression. Mechanistically, circFAM65A drives CANX phase separation, which recruits FAM134B into condensates and blocks its interaction with LC3, thereby inhibiting ER‐phagy and HCC progression. Importantly, targeted circFAM65A delivery represents a potential therapeutic strategy for HCC.

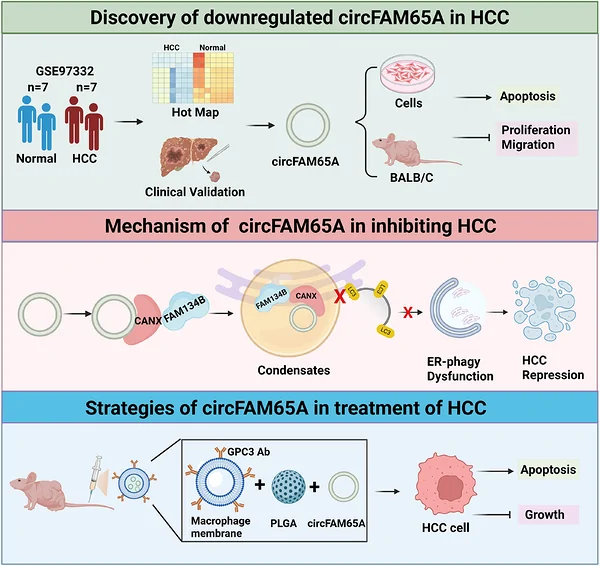

## 1 Introduction

Hepatocellular carcinoma (HCC) is the third leading cause of cancer‐related death, and its incidence is still increasing globally [1]. Although advancements in surgical, locoregional, and systemic therapies have improved patient outcomes, the overall prognosis remains poor, largely because most patients are diagnosed at advanced or metastatic stage [2]. Thus, further investigation of the mechanisms underlying HCC progression is essential for the development of novel therapeutic strategies.

Autophagy, a lysosome‑mediated degradation process, plays a key role in maintaining homeostasis in response to nutrient deprivation and various stress conditions [3]. ER‐phagy is a form of selective autophagy induced by nutrient starvation or the unfolded protein response (UPR) [4]. It is well established that ER‐phagy serves as a protective mechanism in tumor cells. Tumor cells upregulate autophagy to survive microenvironmental stress by selectively degrading damaged organelles and misfolded proteins, thereby recycling macromolecules and amino acids for biosynthetic metabolism and energy production [5, 6]. A growing number of studies have focused on the role of autophagy in HCC progression and its potential as a therapeutic target [7, 8]. In particular, several studies have demonstrated that autophagy contributes to HCC growth, metastasis, and therapeutic resistance [9, 10, 11]. However, the key regulatory molecules and mechanisms of autophagy in HCC remain poorly understood.

Liquid‐liquid phase separation (LLPS) is a biophysical process by which certain proteins and nucleic acids condense into liquid‐like, membraneless organelles, providing a general mechanism for the spatiotemporal control of diverse biological activities within cells [12]. Notably, dysregulation of LLPS is increasingly recognized as a key driver of tumorigenesis [13, 14]. Recent studies have shown that LLPS is also extensively involved in autophagy regulation, including the autophagosome initiation and formation, substrate recognition and recruitment, and transcriptional control of autophagy‐related genes [15]. Further investigation is required to elucidate the link between LLPS and autophagy in HCC progression, which may facilitate the development of novel therapeutic strategies.

Circular RNAs (circRNAs) have been shown to exert tumor‐promoting or tumor‐suppressive functions through diverse mechanisms, including miRNA sequestration, protein scaffolding, and serving as templates for translation [16, 17]. In addition to their canonical functions, several circRNAs have been shown to regulate tumor progression by modulating protein LLPS [18, 19, 20, 21]. For instance, circVAMP3 promotes the formation of stress granules by driving phase separation of CAPRIN1, which suppresses c‐Myc translation and inhibits HCC progression [20]. Currently, research on circRNA‐driven LLPS in HCC remains limited, and the mechanisms by which circRNAs regulate the dynamic assembly of tumor‐associated LLPS and drive tumor progression warrant further investigation.

In this study, we identified circFAM65A as a novel circRNA that was markedly downregulated in HCC. Overexpression of circFAM65A significantly inhibited HCC progression both in vitro and in vivo. Mechanistically, we found that circFAM65A was associated with CANX and suppressed CANX‐enhanced ER‐phagy and HCC progression. Notably, CANX exhibits a propensity to form condensates in HCC cells and in vitro, and this process was enhanced by circFAM65A. CircFAM65A‐associated CANX condensates were accompanied by FAM134B recruitment, which reduced the FAM134B‐LC3 interaction and suppressed ER‐phagy.

We also developed a biomimetic cell membrane‐coated nanoparticle (PLGA/circFAM65A@M‐3E11) for circFAM65A delivery and demonstrated promising anti‐tumor efficacy in nude mice bearing HCC xenografts, suggesting a potential therapeutic strategy.

## 2 Results

### 2.1 CircFAM65A is Downregulated in HCC and its Overexpression Suppresses HCC Progression In Vitro and In Vivo

To identify dysregulated circRNAs in HCC, we analyzed circRNA microarray datasets (GSE97332). Among the candidates, we identified a circRNA (hsa_circ_0000708) that was significantly downregulated in HCC (Figure 1a). It is derived from intron 1 of the FAM65A gene and was thus named circFAM65A; its closed‐loop structure was validated by Sanger sequencing and RT‐PCR using convergent and divergent primers (Figure 1b and Figure S1a). The downregulation of circFAM65A was further confirmed by quantitative real‐time PCR (qRT‐PCR) and fluorescence in situ hybridization (FISH) in HCC tissues and cell lines (Figure 1c,d and Figure S1b). Like most circRNAs, circFAM65A was more resistant to RNase R or actinomycin D treatment than its linear form (Figure S1c,d). We then examined the subcellular distribution of circFAM65A by FISH and interestingly observed that it formed cytoplasmic puncta in HCC cells (Figure 1e). We also used the MS2‐GFP system to label circFAM65A and confirmed its punctate distribution in HCC cells by live‐cell imaging (Figure S1e,f).

**FIGURE 1:**
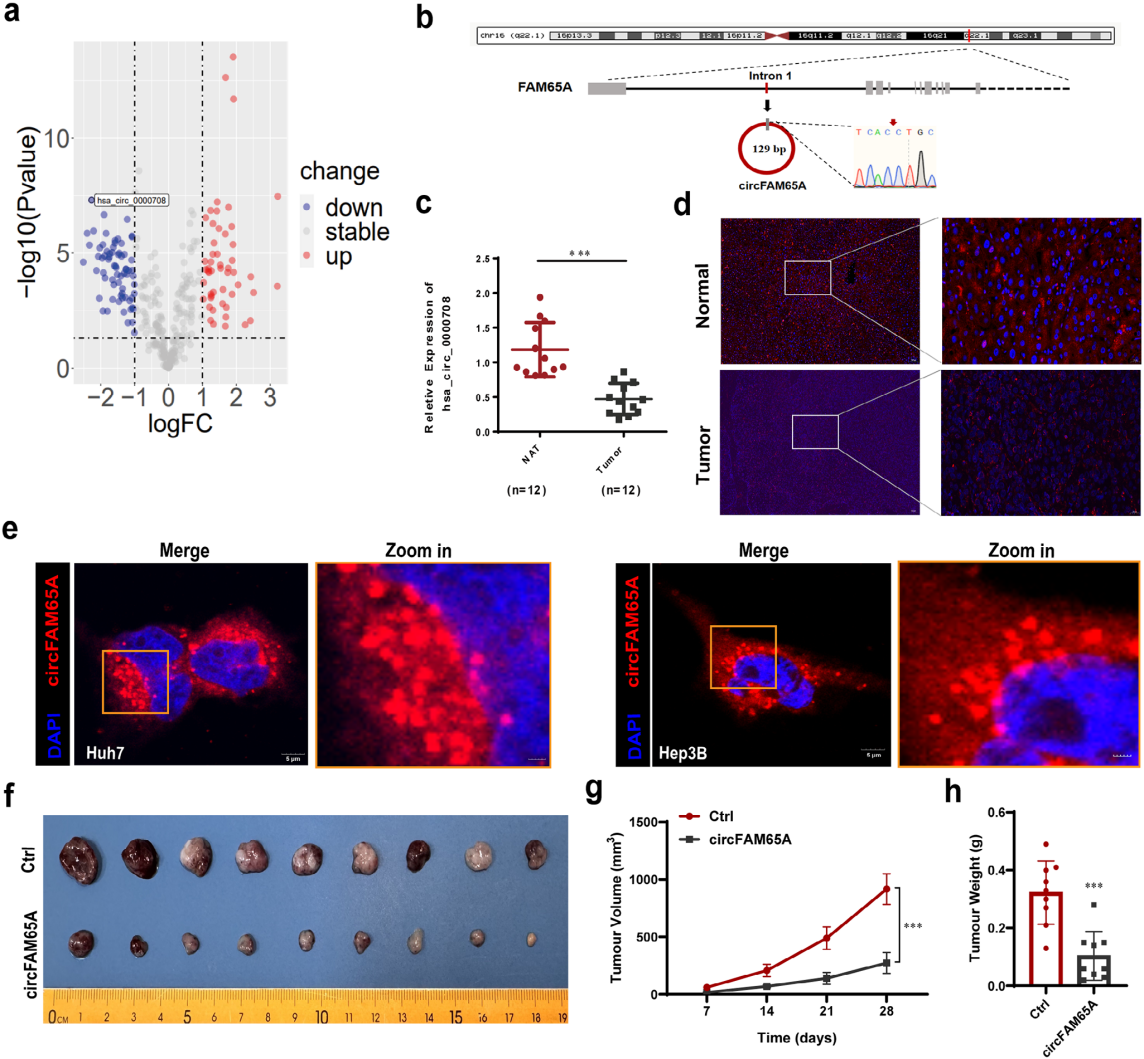
CircFAM65A is downregulated in HCC and its overexpression suppresses HCC tumor growth in vivo. (a) Volcano plot showing differentially expressed circRNAs between HCC and adjacent normal tissues. (b) Schematic illustration of the genomic location and back‐splicing of circFAM65A, with the back‐splice junction validated by Sanger sequencing. (c) RT‐qPCR analysis of circFAM65A expression in 12 pairs of HCC and adjacent normal tissues. (d) FISH images showing circFAM65A expression in representative HCC and matched normal tissues (n = 3 patients per group). Scale bar, 10 µm. (e) Subcellular localization of circFAM65A in HCC cells detected by FISH. Scale bar, 5 µm. (f–h) Subcutaneous tumor images (f), tumor growth curves (g) and tumor weight (h) from circFAM65A‐overexpressing or control Huh7 xenografts. Each group contained 9 mice (n = 9). All microscopy images are representative of n = 3 independent experiments. Data are mean ± s.d. Statistical analysis was performed using paired (c) and unpaired (h) two‐tailed Student's t‐test, or two‐way repeated‐measures ANOVA with Šídák's multiple‐comparisons test (g). ***P < 0.001.

To investigate the biological function of circFAM65A in HCC, we overexpressed it in HepG2 and HCC‐LM3 cells and knocked it down in Huh7 and Hep3B cells, and the overexpression and knockdown efficiencies was verified by qRT‐PCR (Figure S2a,b). As indicated by CCK8, colony formation, and EdU assays, circFAM65A overexpression greatly inhibited the proliferation ability of HCC cells (Figure S2c–e), whereas its knockdown promoted cell proliferation (Figure S2f–h). Transwell and wound healing assays further showed that cell migratory capacity was significantly decreased by circFAM65A overexpression and enhanced by circFAM65A silencing (Figure S2i–l). Moreover, we stably overexpressed circFAM65A in Huh7 cells and subcutaneously injected the cells into nude mice, and then examined its influence on tumor growth in vivo. Results showed that circFAM65A overexpression significantly inhibited tumor growth (Figure 1f–h). Thus, these findings demonstrate that circFAM65A is downregulated in HCC, and its overexpression suppresses cell proliferation and migration in vitro and tumor growth in vivo.

### 2.2 CircFAM65A Associates and Co‐Aggregates With CANX in HCC Cells

Given the punctate distribution of circFAM65A in HCC cells, we hypothesized that it might interact with proximal proteins with a propensity for LLPS. To identify proteins proximal to circFAM65A, we performed HyPro‐MS, a proximity labeling‐based proteomic approach (Figure 2a). According to the MS data, 116 proteins were specifically labeled by circFAM65A, but not by the control, in circFAM65A overexpressing Huh7 cells (Figure S3a). KEGG and Gene Ontology (GO) enrichment analyses revealed that these proteins were enriched in endoplasmic reticulum (ER)‐related biological processes (Figure 2b and Figure S3b). Among these candidates, CANX (calnexin), a member of the ER calcium‐binding protein family, was significantly enriched by circFAM65A (Figure 2c). Notably, sequence analysis revealed that CANX contains intrinsically disordered regions (IDRs) (Figure S3c,d), which are features essential for LLPS [22]. To further validate the interaction between circFAM65A and CANX, we employed the circRNA‐TurboID proximity labeling system and detected the biotinylated proteins by Western blotting (Figure S3e). The results showed that CANX was biotin‐labeled in proximity to circFAM65A (Figure 2d). RNA immunoprecipitation (RIP) assays also supported this interaction (Figure S3f).

**FIGURE 2:**
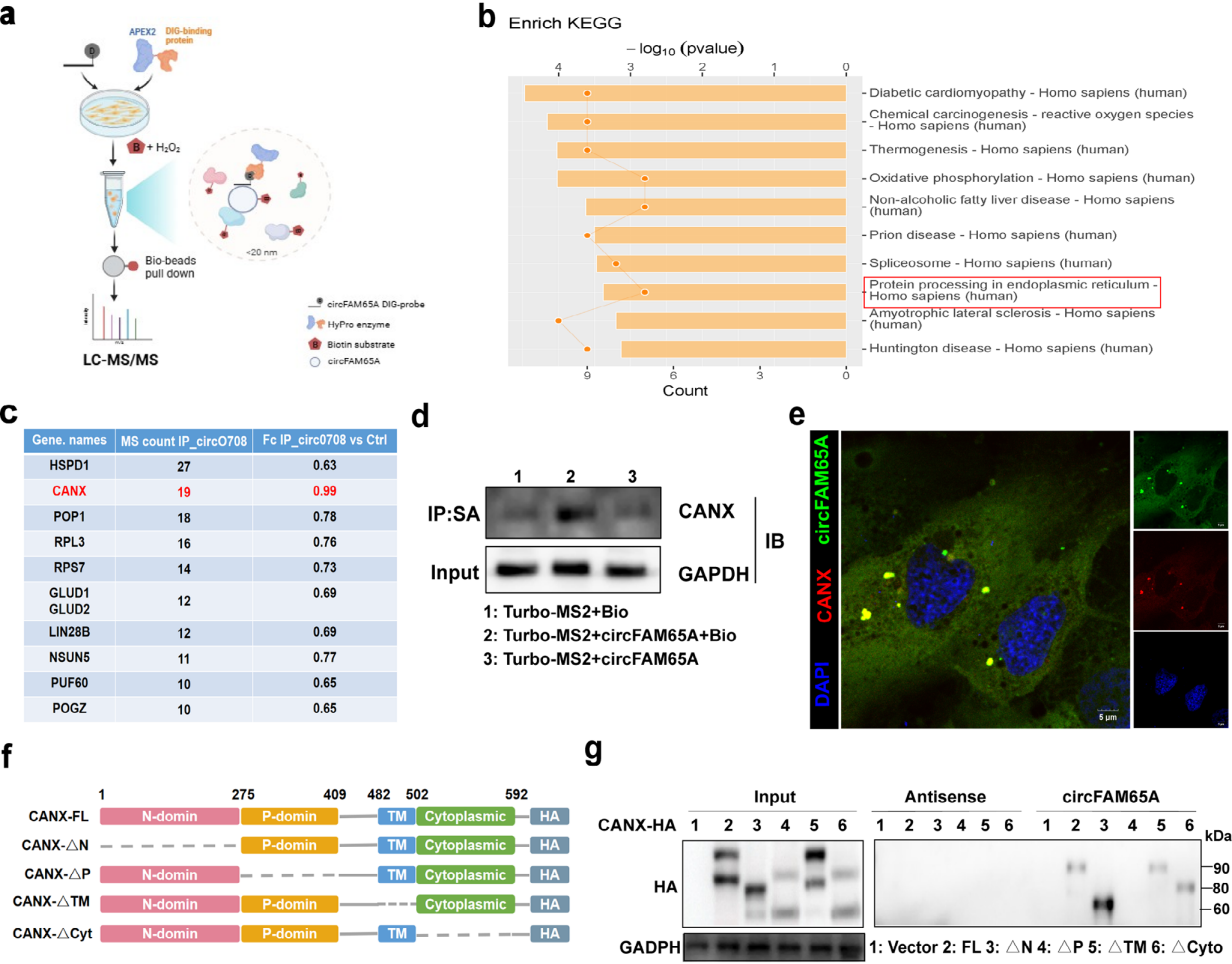
CircFAM65A associates and co‐aggregates with CANX in HCC cells. (a) Schematic representation of the Hypro‐MS analysis. (b) KEGG analysis of circFAM65A‐interacting proteins. (c) Top 10 candidate circFAM65A‐interacting proteins identified by Hypro‐MS. (d) Biotinylated proteins were immunoprecipitated using streptavidin magnetic beads and immunoblotted for CANX. (e) Representative IF images showing co‐aggregation of circFAM65A and CANX in Huh7 cells. Scale bar, 5 µm. (f) Schematic representation of full‐length and truncated CANX constructs. (g) RNA pull‐down assay showing the association between circFAM65A and various CANX truncation mutants in Huh7 cells. All microscopy images and blots are representative of n = 3 independent experiments.

Immunofluorescence (IF) staining of CANX, combined with MS2‐GFP labeling of circFAM65A, showed that CANX and circFAM65A co‐localized in the cytoplasm as condensates (Figure 2e). To further identify the CANX region required for the detected association with circFAM65A, we generated several CANX truncations with different domain deletions (Figure 2f). RNA pull‐down assay revealed that the P domain‐containing region of CANX contributes to its association with circFAM65A (Figure 2g). Furthermore, we verified the co‐localization of different CANX truncations with circFAM65A in live cells. The results showed that deletion of the P domain markedly impaired the formation of circFAM65A/CANX condensates (Figure S3g). These data support an association and co‐aggregation between circFAM65A and CANX in HCC cells.

### 2.3 CANX Promotes ER‐phagy and HCC Progression In Vitro and In Vivo

Next, we evaluated the role of CANX in HCC progression. TCGA database analysis showed that CANX expression was elevated in HCC tissues (Figure 3a), and patients with high CANX expression exhibited poorer overall survival (Figure S4a,b). Western blot and immunohistochemical (IHC) staining confirmed that CANX expression was upregulated in HCC tissues and cell lines (Figure 3b,c and Figure S4c,d). Then, we overexpressed CANX in Hep3B cells and knocked it down in Huh7 cells (Figure S4e,f). CCK8 and colony formation assays revealed that CANX overexpression significantly promoted HCC cell proliferation, while CANX knockdown suppressed it (Figure S4g,h). Wound healing and transwell assays demonstrated that CANX accelerated cell migration (Figure S4i,j). To further investigate its function in vivo, we subcutaneously inoculated CANX knockdown or control Huh7 cells into nude mice, and found that the tumor growth was significantly inhibited in the CANX knockdown group (Figure 3d–f). These results indicated that CANX promotes HCC progression.

**FIGURE 3:**
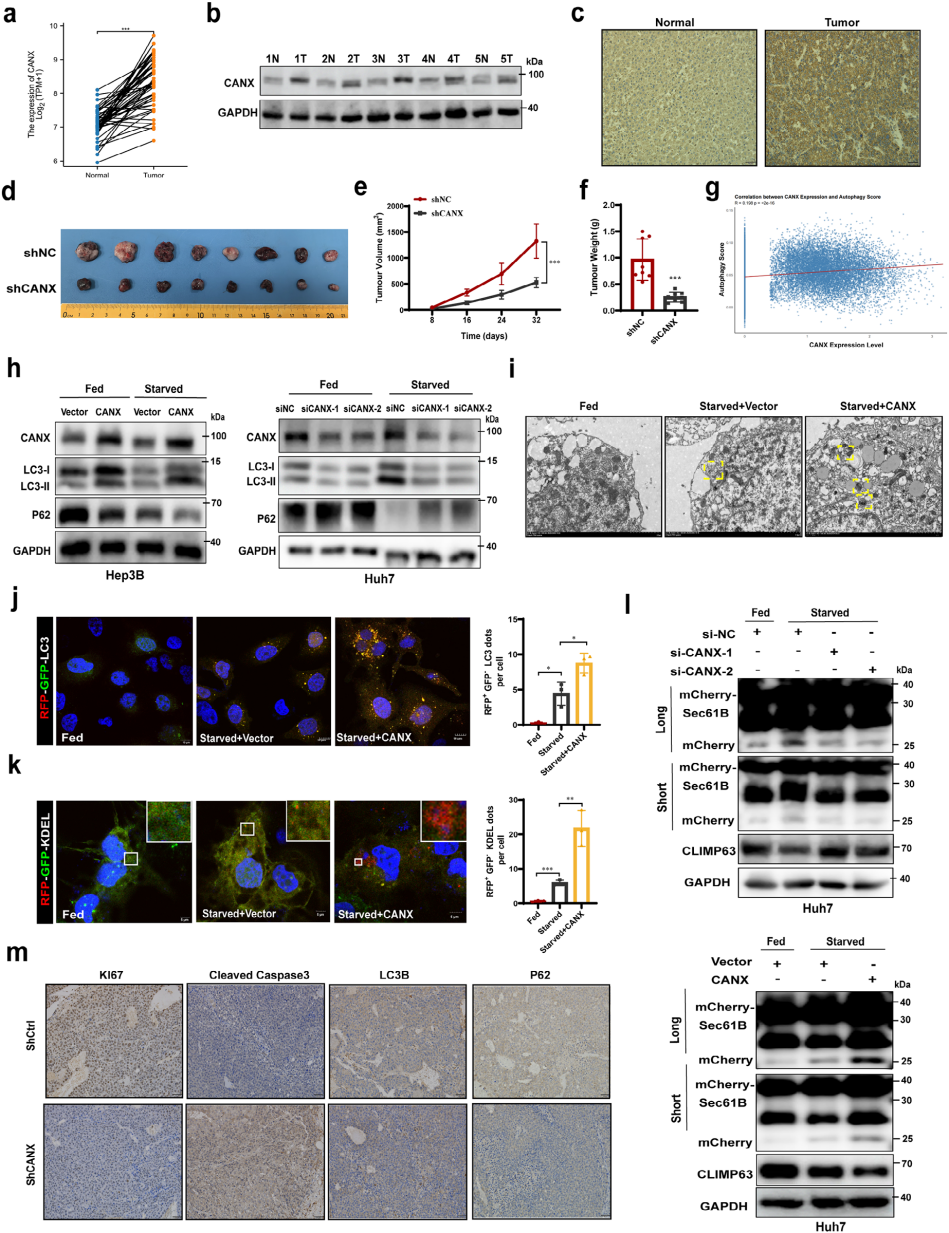
CANX promotes ER‐phagy and HCC progression in vitro and in vivo. (a) Analysis of CANX mRNA expression in tumor and matched adjacent normal tissues using the TCGA HCC cohort. (b) Western blot analysis of CANX protein levels in 5 pairs of HCC tumor tissues (T) and adjacent normal tissues (N). (c) Representative IHC staining of CANX in normal samples and HCC tissues (n = 3 patients per group). Scale bar, 50 µm. (d–f) Subcutaneous tumor images (d), tumor growth curves (e) and tumor weight (f) from CANX‐knockdown and control Huh7 xenografts. Each group contained 8 mice (n = 8). (g) Correlation analysis between CANX expression and autophagy from a single‐cell database. (h) Western blot analysis of CANX, LC3‐II, and p62 expression changes following CANX overexpression or knockdown under normal or EBSS‐treated conditions. (i) TEM images showing autophagosomes (indicated by boxes) in CANX‐overexpressing Huh7 cells after EBSS treatment. Scale bar, 2 µm. (j,k) Representative fluorescence images of autophagic flux using the RFP‐GFP‐LC3 reporter (j) and RFP‐GFP‐KDEL reporter (k) in CANX‐overexpressing Huh7 cells after EBSS treatment. Scale bar, 10 µm. Quantification of RFP^+^GFP^−^ puncta per cell is shown in the right panel. (l) Western blot analysis of free mCherry levels using the mCherry‐Sec61B reporter in CANX‐overexpressing or CANX‐knockdown Huh7 cells after EBSS treatment. (m) IHC staining of Ki‐67, cleaved caspase3, LC3B and p62 in subcutaneous tumor. Scale bar, 50 µm. All microscopy images and blots are representative of n = 3 independent experiments. Data are mean ± s.d. Statistical analysis was performed using unpaired two‐tailed Student's t‐test (f), one‐way ANOVA followed by Tukey's multiple comparisons test (j, k), or two‐way repeated‐measures ANOVA followed by Šídák's multiple comparisons (e). *P < 0.05, **P < 0.01, ***P < 0.001.

Previous studies have shown that CANX can regulate autophagy [23, 24, 25]; however, its specific role and mechanism in HCC remain elusive. We analyzed TCGA data, classified HCC patients into high‐ and low‐autophagy groups, and found that patients with high autophagy had markedly poorer prognosis than those with low autophagy (Figure S5a). IHC staining of LC3B and p62 in HCC and adjacent normal tissues showed that the abundance of these autophagy‐related markers was altered, suggesting a general increase in baseline autophagy in HCC tissues (Figure S5b). These findings suggest that autophagy levels are closely linked to HCC malignancy. To further assess the relationship between CANX and autophagy in HCC, we analyzed the data from single‐cell sequencing (scRNA‐seq) and the TCGA database, and found a strong positive correlation (Figure 3g and Figure S5c,d). Then we examined whether CANX regulates autophagy in HCC cells. Western blot analysis showed that CANX overexpression significantly increased LC3‐II levels and reduced p62 expression, whereas CANX knockdown suppressed it (Figure 3h). We also examined autophagic flux in Huh7 cells treated with the late‐stage autophagy inhibitor chloroquine (CQ), and the results showed that CANX overexpression significantly enhanced LC3‐II accumulation, whereas CANX knockdown had the opposite effect (Figure S5e). Moreover, transmission electron microscopy (TEM) revealed a greater number of autophagosomes in CANX‐overexpressing HCC cells (Figure 3i). Consistent with these findings, RFP‐GFP‐LC3 reporter assay further confirmed that CANX overexpression promoted autophagic flux (Figure 3j).

Given that CANX is an ER‐resident protein, we examined its potential role in ER‐phagy using the RFP‐GFP‐KDEL tandem tagging strategy. Following Earle's Balanced Salt Solution (EBSS) treatment, CANX overexpression increased starvation‐induced ER‐phagy (Figure 3k). We further examined ER‐phagy activation using the mCherry‐Sec61B reporter, which releases mCherry fragments detectable by Western blot upon ER‐phagy‐induced Sec61B digestion [26]. As expected, CANX overexpression increased the level of free mCherry and reduced the abundance of the ER‐resident protein CLIMP‐63, whereas CANX knockdown had the opposite effect (Figure 3l).

ER‐phagy is known to alleviate ER stress and inhibit cellular apoptosis [5]. To determine whether CANX‐mediated ER‐phagy affects apoptosis, we examined apoptosis upon CANX overexpression or knockdown in HCC cells. The results demonstrated that CANX overexpression reduced apoptosis, whereas its knockdown promoted it (Figure S4k). Additionally, IHC analyses in subcutaneous tumor models showed that CANX deficiency decreased autophagy‐related marker levels and increased cleaved caspase‐3 levels (Figure 3m). Collectively, these findings demonstrate that CANX promotes ER‐phagy while suppressing apoptosis in HCC both in vitro and in vivo.

### 2.4 CircFAM65A Enhances CANX Condensate Formation in HCC Cells and In Vitro

Since CANX contains IDRs, we first examined its condensate propensity in HCC cells. Both endogenous CANX and exogenously expressed CANX‐mNeonGreen formed distinct droplets in Huh7 and Hep3B cells (Figure 4a and Figure S6a). Fluorescence recovery after photobleaching (FRAP) assay confirmed their high dynamic nature (Figure 4b). In vitro, purified CANX‐EGFP protein formed liquid droplets that exhibited fusion behavior (Figure S6b–d), and FRAP further confirmed their liquid‐like nature (Figure 4c). Droplet formation was facilitated by PEG8000 and disrupted by 1,6‐hexanediol in a concentration‐dependent manner (Figure 4d,e). Droplet formation was also modulated by protein concentration and salt concentration (Figure 4f). Sequence analysis revealed two IDRs in CANX: IDR1 (258‐358 aa) and IDR2 (524‐592 aa). To map the regions responsible for condensate formation, we generated CANX mutants lacking IDR1, IDR2, or both (Figure S6e). Deletion of IDR2, either alone or in combination with IDR1, markedly reduced CANX puncta in HCC cells and droplet formation in vitro (Figure S6f,g). Collectively, these results suggest that CANX has the propensity to undergo condensation in an IDR2‐dependent manner.

**FIGURE 4:**
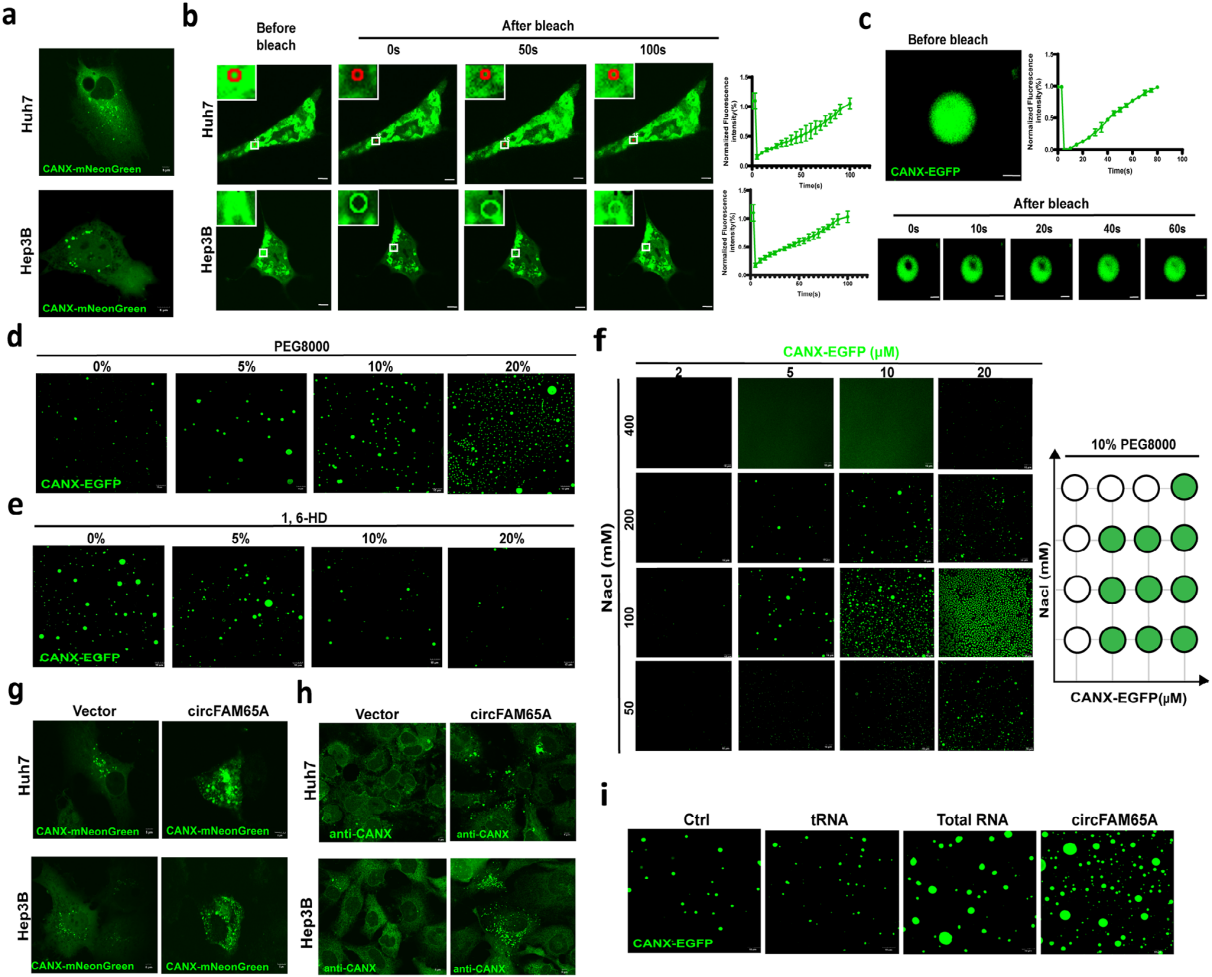
CircFAM65A enhances CANX condensate formation in HCC cells and in vitro. (a) Representative confocal imaging showing punctate condensates of CANX‐mNeonGreen in HCC cells. Scale bar, 5 µm. (b,c) Representative images and fluorescence recovery curves of CANX‐mNeonGreen in HCC cells (b) and of purified CANX‐EGFP in vitro (c) after laser photobleaching. Scale bar, 5 µm. (d,e) In vitro phase separation of purified CANX‐EGFP under different concentrations of PEG8000 (d) and 1,6‐hexanediol (e). Scale bar, 15 µm. (f) Phase separation of purified CANX‐EGFP at varying protein and NaCl concentrations in the presence of 10% PEG8000. Scale bar, 15 µm. (g,h) Representative confocal imaging of CANX‐mNeonGreen condensates (g) or IF staining of endogenous CANX (h) in control and circFAM65A‐overexpressing HCC cells. Scale bar, 5 µm. (i) In vitro phase separation of purified CANX‐EGFP in the presence of different RNAs. Scale bar, 10 µm. All microscopy images are representative of n = 3 independent experiments.

Given that circRNAs can modulate phase separation of their interacting proteins [18, 19, 20, 21], we asked whether circFAM65A influences CANX condensate formation. CircFAM65A overexpression did not affect the expression of CANX (Figure S6h,i), but markedly increased CANX‐mNeonGreen puncta in HCC cells (Figure 4g), a finding corroborated by IF staining of endogenous CANX (Figure 4h). In vitro, both synthetic circFAM65A and total RNA isolated from HCC cells promoted CANX droplet formation, whereas tRNAs did not (Figure 4i). RNase A, but not RNase R, abrogated this effect, suggesting that circular RNA may promote CANX condensate formation in this context (Figure S6j). To exclude the possibility that CANX condensates represented non‐specific aggregation caused by ER stress or exposed hydrophobic transmembrane domains, we separately treated Huh7 cells with 1 µM ER stress activator thapsigargin, or 5% 1,6‐hexanediol or 0.01% Tween 20 in the presence of circFAM65A overexpression. Thapsigargin and Tween 20 did not obviously alter the number of CANX condensates induced by circFAM65A, whereas 1,6‐hexanediol markedly reduced them (Figure S6k,l). These results suggest that circFAM65A promoted CANX condensate formation in cells, although these treatments did not establish LLPS as its sole basis. Additionally, no co‐localization was detected between CANX punctate structures and p62‐positive aggregates, indicating that these CANX puncta are not autophagy‐targeted protein aggregates (Figure S6m). Collectively, these data support that circFAM65A enhances CANX condensate formation.

### 2.5 CircFAM65A Attenuates CANX‐Promoted ER‐phagy and HCC Progression

To determine whether circFAM65A regulates CANX‐induced ER‐phagy, we first examined its effect on general autophagy. RNA‐seq analysis revealed that circFAM65A overexpression suppressed autophagy‐related genes in HCC cells (Figure S7a). This was further validated by Western blot and RFP‐GFP‐LC3 reporter assay, showing that circFAM65A overexpression inhibited autophagy, while its knockdown had the opposite effect (Figure S7b–d). Consistently, mCherry‐Sec61B and RFP‐GFP‐KDEL reporter assays confirmed that circFAM65A overexpression suppressed ER‐phagy (Figure S7e,f). Moreover, flow cytometry assay revealed that circFAM65A significantly increased apoptosis of HCC cells (Figure S7g). IHC staining of xenograft tumors showed increased p62 and decreased LC3B abundance in the circFAM65A‐overexpressing group, consistent with changes in autophagy‐related markers (Figure S7h).

We next investigated whether circFAM65A attenuates CANX‐promoted ER‐phagy, and whether this effect depends on the circFAM65A‐CANX interaction and the condensate property of CANX. Rescue assays showed that exogenous co‐expression of circFAM65A with CANX^FL^ effectively reversed CANX‐promoted ER‐phagy, whereas co‐expression with the CANX^ΔP^ or CANX^ΔIDR2^ failed to do so (Figure 5a–d). Consistently, functional assays showed that co‐expression with CANX^FL^, but not with CANX^ΔP^ or CANX^ΔIDR2,^ reversed CANX‐promoted cell proliferation and migration as well as the anti‐apoptotic effect of CANX (Figure S8a–c). These findings were recapitulated in vivo: CANX overexpression accelerated tumor growth, an effect abrogated by co‐expression of circFAM65A (Figure 5e–g). IHC analyses further confirmed that circFAM65A overexpression reversed the changes in autophagy and apoptosis marker abundance induced by CANX in vivo (Figure 5h).

**FIGURE 5:**
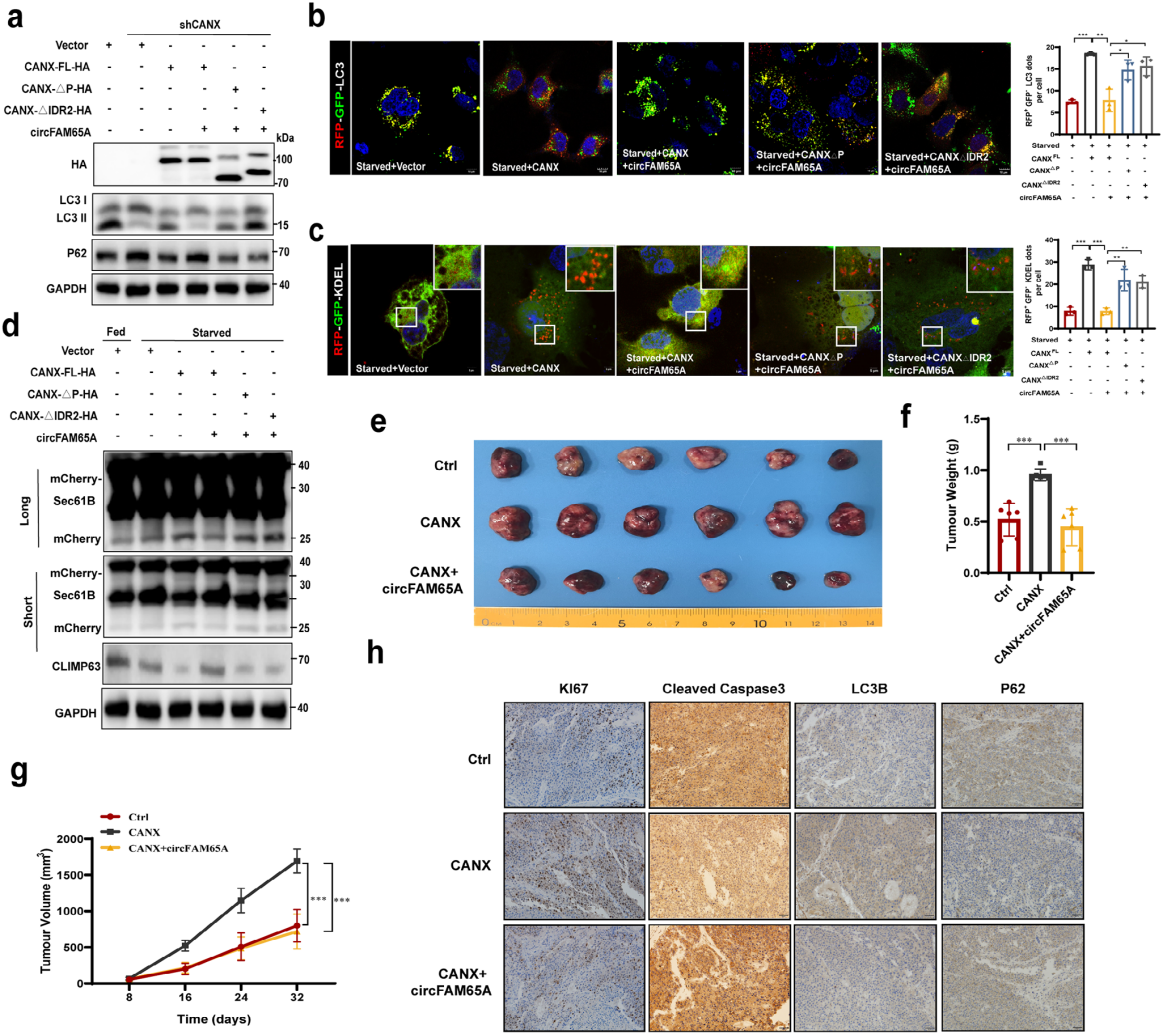
CircFAM65A attenuates CANX‐promoted ER‐phagy and HCC progression. (a) Western blotting shows the changes in LC3‐II and p62 expression in CANX‐knockdown Huh7 cells following overexpression of CANX^FL^ alone, or co‐transfection of circFAM65A with CANX^FL^, CANX^ΔP^ or CANX^ΔIDR2^. (b,c) Representative images of RFP‐GFP‐LC3 reporter (Scale bar, 10 µm) (b) and RFP‐GFP‐KDEL reporter (Scale bar, 5 µm) (c) in Huh7 cells after CANX^FL^ overexpression or circFAM65A co‐transfection with CANX^FL^, CANX^ΔP^ or CANX^ΔIDR2^. Quantification of RFP^+^GFP^−^ puncta per cell is shown in the right panel. (d) Western blot analysis of free mCherry levels using the mCherry‐Sec61B reporter in Huh7 cells with indicated transfections. (e–g) Representative images of subcutaneous tumors (e), tumor weight (f) and tumor growth curves (g) from CANX‐overexpressing Huh7 xenografts with or without circFAM65A co‐overexpression. Each group contained 6 mice (n = 6). (h) IHC staining of Ki‐67, cleaved caspase3, LC3B and p62 in subcutaneous tumor. Scale bar, 50 µm. All microscopy images and blots are representative of n = 3 independent experiments. Data are mean ± s.d. Statistical analysis was performed using one‐way ANOVA followed by Tukey's multiple comparisons test (b, c, f), or two‐way repeated‐measures ANOV followed by Šídák's multiple‐comparisons test (g). *P < 0.05, **P < 0.01, ***P < 0.001.

ER‐phagy has been proposed as a critical mechanism to alleviate ER stress and prevent apoptosis. Notably, recent studies have demonstrated that CANX deficiency can directly induce ER stress [27]. We therefore assessed whether circFAM65A could modulate CANX‐mediated inhibition of ER stress. Co‐expression of circFAM65A with CANX^FL^ effectively reversed the CANX‐induced suppression of ER stress following thapsigargin (TG) treatment, whereas co‐expression with CANX^ΔP^ or CANX^ΔIDR2^ failed to exert this effect (Figure S8d). Overall, these data suggest that circFAM65A attenuates CANX‐promoted ER‐phagy and HCC progression, at least in part through its interaction with CANX and effects associated with CANX condensation.

### 2.6 CircFAM65A/CANX Condensates Sequester FAM134B and Inhibit ER‐phagy

Next, we sought to explore how circFAM65A regulates CANX function through condensates. ER‐phagy receptors mediate the connection between ER membranes and autophagosomes [4]. Screening for CANX‐interacting receptors identified FAM134B as a direct partner (Figure S9a–c). However, circFAM65A did not substantially affect CANX‐FAM134B binding (Figure S9d). Given that circFAM65A promotes CANX condensate formation, we investigated whether FAM134B is incorporated into circFAM65A/CANX condensates. Intriguingly, FAM134B was recruited into CANX condensates only when circFAM65A was present, and CANX alone failed to recruit FAM134B (Figure 6a). In vitro, FAM134B‐mCherry did not form droplets either alone or in combination with CANX, but was incorporated into CANX‐EGFP droplets when circFAM65A was added (Figure 6b and Figure S9e). Cellular and in vitro FRAP assays confirmed the molecular dynamics of CANX and FAM134B in droplets upon the addition of circFAM65A (Figure 6c,d). Domain mapping revealed that the TM domain of CANX is essential for FAM134B recruitment, as its deletion abolished FAM134B incorporation into condensates in circFAM65A‐overexpressing cells (Figure 6e,f). Consistently, the condensate property of CANX is also required for this process (Figure 6f). Collectively, these findings suggest that CANX interacts with FAM134B and sequesters it within circFAM65A/CANX condensates.

**FIGURE 6:**
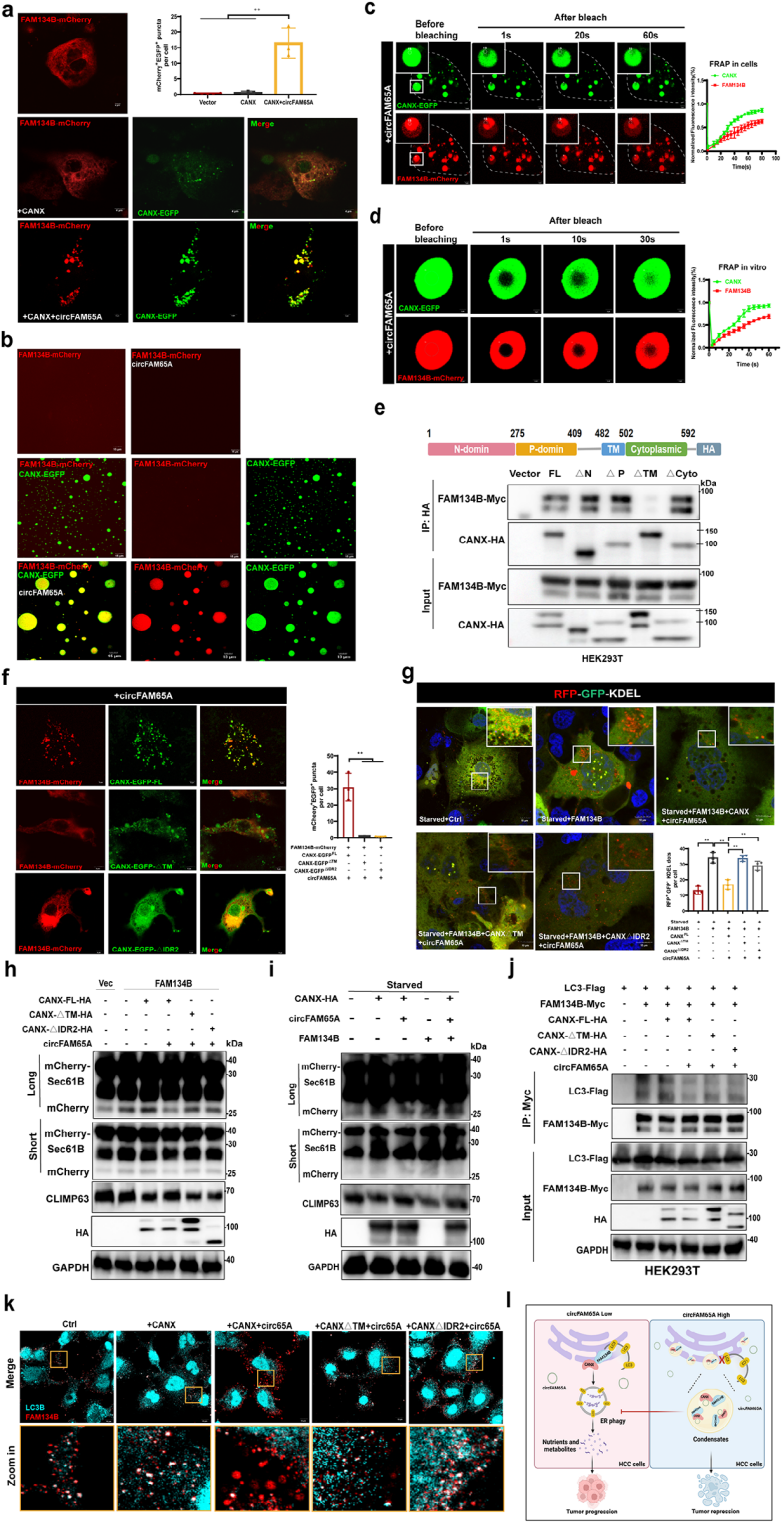
CircFAM65A/CANX condensates sequester FAM134B and inhibit ER‐phagy. (a) Representative confocal imaging of HCC cells expressing FAM134B‐mCherry alone, FAM134B‐mCherry with CANX‐EGFP, or FAM134B‐mCherry with CANX‐EGFP and circFAM65A. Scale bar, 5 µm. Quantification of mCherry^+^EGFP^+^ puncta per cell is shown in the right panel. (b) In vitro droplet formation assay of purified FAM134B‐mCherry alone, FAM134B‐mCherry with circFAM65A, FAM134B‐mCherry with CANX‐EGFP, or FAM134B‐mCherry with CANX‐EGFP and circFAM65A. Scale bar, 15 µm. (c, d) Representative FRAP images and fluorescence recovery quantitative analysis of CANX‐EGFP and FAM134B‐mCherry with circFAM65A treatment in live Huh7 cells (c) or in vitro system (d). Scale bar, 3 µm. (e) Co‐IP analysis showing the interactions between each CANX‐HA truncation and FAM134B‐Myc. (f) Representative confocal imaging of cells expressing FAM134B‐mCherry with CANX‐EGFP, CANX^ΔTM^‐EGFP, CANX^ΔIDR2^‐EGFP in circFAM65A‐overexpressing Huh7 cells. Scale bar, 5 µm. Quantification of mCherry^+^EGFP^+^ puncta per cell is shown in the right panel. (g) Representative ER‐phagy flux images of RFP‐GFP‐KDEL reporter in FAM134B‐overexpressing Huh7 cells, co‐expressing circFAM65A and CANX^FL^, CANX^ΔTM^ or CANX^ΔIDR2^. Scale bar, 10 µm. Quantification of RFP^+^GFP^−^ puncta per cell is shown in the right panel. (h) Western blot analysis of free mCherry levels using the mCherry‐Sec61B reporter in FAM134B‐overexpressing Huh7 cells co‐expressing CANX^FL^, or co‐expressing circFAM65A and CANX^FL^, CANX^ΔTM^ or CANX^ΔIDR2^. (i) Western blot analysis of free mCherry levels using the mCherry‐Sec61B reporter in Huh7 cells with CANX, circFAM65A, and FAM134B co‐overexpression. (j) Co‐IP assay showing the interactions between FAM134B‐Myc and LC3‐Flag in HEK293T cells co‐expressing CANX^FL^, or co‐expressing circFAM65A and CANX^FL^, CANX^ΔTM^, or CANX^ΔIDR2^. (k) Representative fluorescence imaging of LC3B and FAM134B staining by IF in Huh7 cells with indicated transfections. (l) Working model illustrating the hypothesized mechanism: in circFAM65A‐overexpressing HCC cells, circFAM65A associates with CANX and promotes CANX condensate formation. These condensates recruit FAM134B and block its interaction with LC3, inhibiting ER‐phagy and HCC progression. All microscopy images and blots are representative of n = 3 independent experiments. Data are mean ± s.d. Statistical analysis was performed using one‐way ANOVA followed by Tukey's multiple comparisons test (a, f, g). **P < 0.01.

To determine whether circFAM65A/CANX condensates inhibit ER‐phagy by sequestering FAM134B, we co‐expressed circFAM65A with CANX or CANX^ΔTM^, CANX^ΔIDR2^ in FAM134B‐expressing HCC cells. RFP‐GFP‐KDEL and mCherry‐Sec61B assays showed that co‐expression of CANX and circFAM65A, but not CANX^ΔTM^/circFAM65A or CANX^ΔIDR2^/circFAM65A, blocked FAM134B‐induced ER‐phagy (Figure 6g,h). Importantly, FAM134B expression rescued the ER‐phagy defect caused by circFAM65A/CANX co‐expression (Figure 6i). We next investigated whether circFAM65A/CANX co‐expression affects FAM134B‐mediated autophagosome tethering. Starvation enhanced CANX‐FAM134B binding, but circFAM65A did not alter this interaction (Figure S9f). Notably, FAM134B‐LC3 interaction was markedly reduced upon circFAM65A/CANX co‐expression, but not in CANX^ΔTM^/circFAM65A or CANX^ΔIDR2^/circFAM65A co‐expression groups (Figure 6j). Consistently, IF staining showed that CANX overexpression promoted FAM134B‐LC3 colocalization, whereas circFAM65A/CANX co‐expression disrupted this colocalization and increased FAM134B puncta (Figure 6k). This disruptive effect was not observed in the CANX^ΔTM^/circFAM65A or CANX^ΔIDR2^/circFAM65A co‐expression groups (Figure 6k). Together, these findings suggest that circFAM65A overexpression might suppress ER‐phagy and HCC progression by promoting CANX condensate formation and the recruitment of FAM134B to these condensates, reducing the FAM134B‐LC3 interaction (Figure 6l).

### 2.7 A Biomimetic Membrane‐Coated Nanoparticle Enables Targeted Delivery of circFAM65A and Effectively Suppresses HCC Growth in Vivo

Given that circFAM65A attenuates CANX‐promoted ER‐phagy and HCC progression, we hypothesized that circFAM65A delivery might serve as a therapeutic strategy for HCC. To this end, we developed a biomimetic nanodelivery system (PLGA/circFAM65A@M‐3E11) comprising circFAM65A‐loaded PLGA nanoparticles coated with macrophage membranes displaying an anti‐GPC3 scFv intended to enhance tumor targeting (Figure 7a) [28]. Membrane coating was verified by scanning electron microscopy (SEM) and TEM (Figure 7b), and further confirmed by size distribution and zeta potential measurements (Figure S10a,b).

**FIGURE 7:**
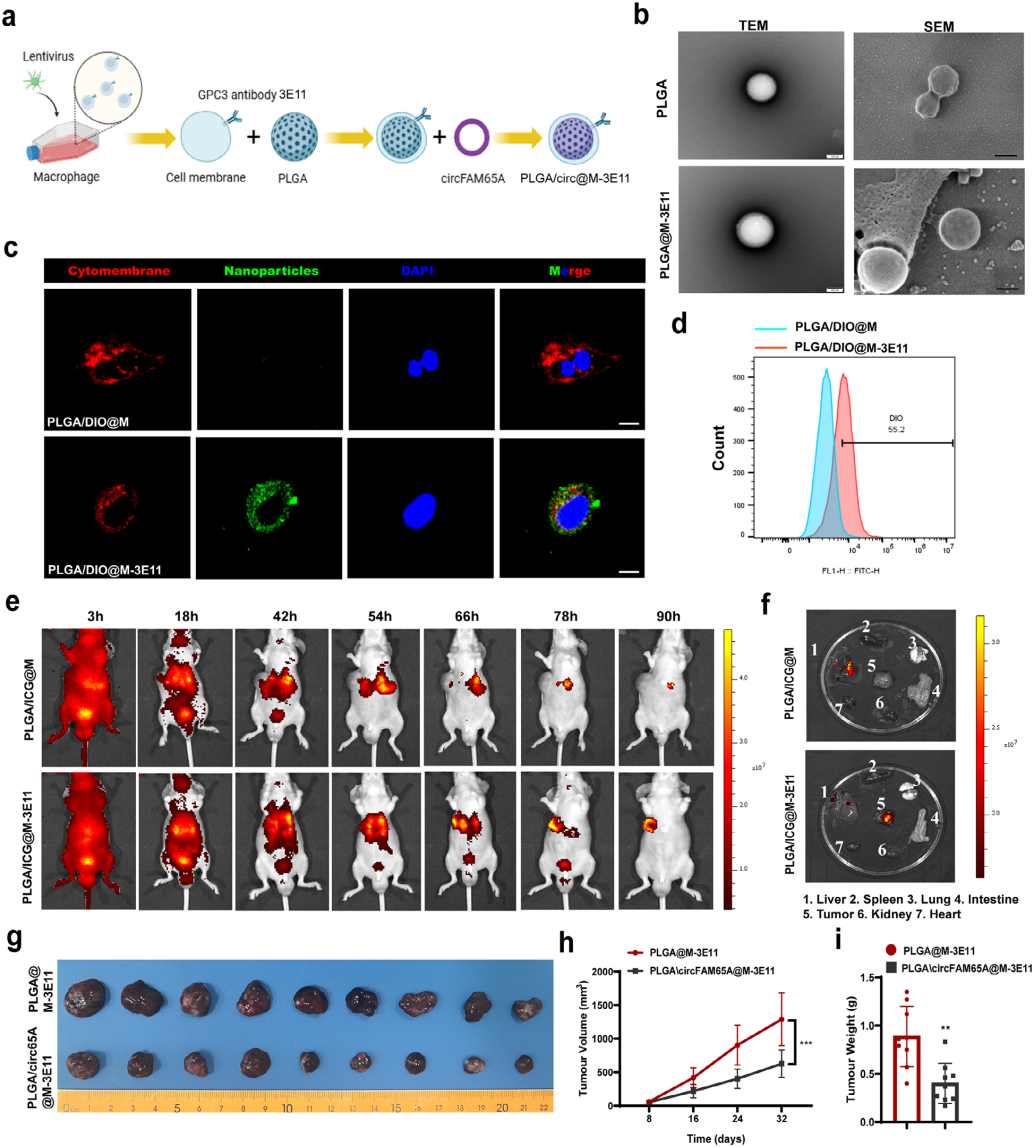
A macrophage membrane‐coated nanoparticle enables targeted delivery of circFAM65A and effectively suppresses HCC growth in vivo. (a) Schematic illustration of the preparation of PLGA/circFAM65A@M‐3E11 nanoparticles. (b) TEM and SEM images showing the morphology of PLGA and PLGA@M‐3E11 nanoparticles. Scale bar, 100 nm. (c,d) Representative confocal imaging (c) and flow cytometry analysis (d) of nanoparticle binding to the HCC cell membrane. Scale bar, 5 µm. (e,f) Representative NIR images of subcutaneous HCC tumors at indicated times after injection (e) and dissected organs from HCC tumor‐bearing mice (f). Each group contained 3 mice (n = 3). (g–i) Subcutaneous tumor images (g), tumor growth curves (h) and tumor weight (i) following treatment with the indicated nanoparticles. Each group contained 9 mice (n = 9). All microscopy images are representative of n = 3 independent experiments. Data are mean ± s.d. Statistical analysis was performed using unpaired two‐tailed Student's t‐test (i), or two‐way repeated‐measures ANOVA followed by Šídák's multiple‐comparisons test (h). **P < 0.01 and ***P < 0.001.

Subsequently, we evaluated cellular uptake and tumor accumulation of fluorescently labeled biomimetic nanoparticles (PLGA/DIO@M‐3E11 or PLGA/ICG@M‐3E11) in vitro and in vivo. Confocal imaging and flow cytometry revealed that PLGA/DIO@M‐3E11 was more efficiently targeted to HCC cells and taken up via endocytosis (Figure 7c,d and Figure S10c). In subcutaneous HCC xenografts, PLGA/ICG@M‐3E11 showed markedly enhanced tumor accumulation compared with PLGA/ICG@M, as demonstrated by whole‐body near‐infrared (NIR) imaging (Figure 7e). Ex vivo imaging of isolated organs further showed greater accumulation in tumor tissue under the conditions tested (Figure 7f). In vitro‐synthesized circFAM65A was then loaded into PLGA@M‐3E11 nanoparticles (Figure S10d,e), and the anti‐tumor efficacy of PLGA/circFAM65A@M‐3E11 was evaluated in a subcutaneous HCC xenograft model (Figure S10f). Successful delivery was indicated by increased circFAM65A levels in tumor tissues (Figure S10g). As expected, PLGA/circFAM65A@M‐3E11 treatment significantly reduced tumor volume and weight compared to the control group (Figure 7g–i), accompanied by decreased proliferation and increased apoptosis, as shown by IHC staining (Figure S10h). Importantly, PLGA/circFAM65A@M‐3E11 treatment caused no detectable changes in serum biochemical parameters or obvious histopathological changes in major organs (Figure S10i,j). Collectively, these results demonstrate the potent anti‐tumor activity of PLGA/circFAM65A@M‐3E11 and support its potential as a therapeutic strategy for HCC.

## 3 Discussion

The relationship between circRNAs and autophagy in HCC remains largely unexplored, and it is not well understood whether or how circRNAs modulate autophagy to affect HCC progression. In this study, we identified circFAM65A as a significantly downregulated circRNA in HCC, and confirmed its anti‐tumor effect in vitro and in vivo. Mechanistically, circFAM65A interacted with CANX and enhanced CANX condensate formation. The circFAM65A/CANX condensates recruited FAM134B, accompanied by reduced FAM134B‐LC3 interaction and ER‐phagy. These findings suggest a potential mechanism by which circFAM65A inhibits HCC progression. Furthermore, we developed a biomimetic membrane‐coated nanoparticle that enables delivery of circFAM65A and effectively suppresses HCC growth in vivo. This work uncovers an uncharacterized mechanism by which circFAM65A/CANX condensates regulate autophagy and HCC progression, offering a novel therapeutic strategy for HCC.

It is widely accepted that autophagy exerts tumor‐suppressive effects during cancer initiation by reducing tissue damage and inflammation. However, as tumors progress, microenvironmental stress can enhance autophagy, which subsequently supports tumor cell survival and adaptation [29]. Several lines of evidence have linked autophagy to HCC progression. For instance, lipid deprivation or ER stress induces ADSL phosphorylation, facilitating its binding to Beclin‐1 and disrupting the BCL2‐Beclin‐1 interaction, which promotes autophagy and liver tumor growth [30]. Autophagy also promotes HCC cell invasion by enhancing cancer cell colonization and activating epithelial‐mesenchymal transition (EMT) [10, 31], and contributes to the acquisition and maintenance of drug resistance in HCC [9, 11, 32]. However, the regulatory mechanisms of autophagy in HCC remain poorly understood.

Here, we identified CANX, an ER transmembrane protein involved in protein folding and quality control [33], as an interacting partner of circFAM65A. Studies have shown that CANX regulates autophagy. For example, CANX increases MAPK activity and upregulates BNIP3‐mediated protective mitophagy, thereby promoting glioblastoma progression [24]. Additionally, CANX interacts with and recruits ULK1 to the ER, and loss of CANX inactivates ULK1, suppressing autophagy and contributing to Alzheimer's disease [25]. However, very few studies have investigated the role of CANX in HCC [34, 35]. In this study, we showed that CANX promotes autophagy and HCC progression. Importantly, we further demonstrated that circFAM65A interacts with CANX and attenuates CANX‐promoted ER‐phagy and HCC progression.

LLPS is a key mechanism for regulating protein function and has been implicated in tumor progression. Phase separation can either promote tumor growth by forming functional condensates or exert tumor‐suppressive effects by sequestering or degrading oncogenic proteins [13, 14]. Multivalent interactions between proteins, or between proteins and nucleic acids, generally drive LLPS [36]. RNA has been shown to modulate the LLPS of certain proteins [37, 38, 39, 40]. CircRNAs also play an important role in LLPS. For instance, circASH2 promotes YBX1 LLPS to augment TPM4 transcripts decay, which alters tumor cytoskeleton structure and inhibits HCC metastasis [18]; circCREIT inhibits stress granule assembly by destabilizing PKR and overcomes doxorubicin resistance in triple‐negative breast cancer (TNBC) [21]. However, the interplay between circRNAs and LLPS in HCC remains unclear. Our findings showed that CANX has the propensity to form dynamic condensation, and that circFAM65A promotes this process. These findings provide mechanistic insights into how circRNAs can modulate phase separation to influence tumor progression.

LLPS is also critical for autophagy initiation and regulation [15]. For example, ER Ca^2^^+^ transients upon autophagy stimulation trigger FIP200 LLPS and autophagosome initiation site formation on the ER [41], and CaMKIIβ phosphorylation of FIP200 further promotes this process, inducing autophagosome formation [42]. Coronavirus ORF8 undergoes condensation with p62 and the ORF8/p62 condensates inhibit ER‐phagy by sequestering FAM134B and ATL3, thereby enhancing viral replication [26]. While LLPS has been implicated in ER‐phagy, the underlying mechanism remains elusive. Emerging evidence links circRNAs to LLPS in tumor autophagy. For example, circMPP6 scaffolds the interaction between MEX3A and processing body (PB) proteins to promote PB aggregation, thereby inhibiting autophagy and promoting colorectal cancer progression [19], while circSPECC1 facilitates ATG4B phase separation and degradation to inhibit autophagy in gastric cancer [43]. Similarly, circMTUS1 regulates PABPC1 LLPS to inhibit SG formation and autophagy, thereby attenuating chemotherapy resistance in gastric cancer [44]. In our study, we revealed a previously unrecognized mechanism in HCC, wherein circFAM65A promotes CANX condensate formation to suppress ER‐phagy and HCC progression. Importantly, the circFAM65A‐CANX association and CANX domains linked to condensate formation at least in part contribute to this suppression. CANX has been shown to recruit FAM134B and promote its interaction with LC3 upon misfolded procollagen recognition, delivering the misfolded procollagen to lysosomes [23]. Consistently, we also identified FAM134B as a CANX interactor and showed that circFAM65A/CANX condensates sequester FAM134B, blocking its interaction with LC3. Although these findings support a mechanism involving FAM134B, proof of necessity would require further genetic loss‐of‐function studies followed by re‐expression rescue.

CircRNA is generally stable and may exhibit relatively low immunogenicity [45]. Following the development of the first circRNA vaccine against SARS‐CoV‐2 [46], circRNA‐based therapies have garnered growing interest. Advances in nanomedicine, including lipid nanoparticles, polymeric platforms, and biomimetic membrane‐coated carriers, have enabled both silencing of oncogenic circRNAs and delivery of therapeutic circRNAs. Nonetheless, challenges such as tumor‐specific targeting, immunogenicity, off‐target effects, and biosafety persist [47]. Biomimetic cell membrane‐coated nanodelivery systems exhibit outstanding biocompatibility, improved barrier‐crossing ability, and homologous targeting capability, thereby offering a novel platform for the delivery of therapeutic RNAs, including circRNAs [48]. Given the tumor‐suppressive effects observed with circFAM65A expression, we delivered circFAM65A to HCC cells using a macrophage membrane‐coated nanodelivery system. In vivo, PLGA/circFAM65A@M‐3E11 significantly suppressed tumor growth in subcutaneous HCC xenografts without detectable toxicity. Membrane‐coated biomimetic systems have been reported to reduce phagocytic clearance and facilitate cellular uptake [49, 50]; the contribution of GPC3‐dependent uptake to our formulation remains to be established.

Nevertheless, several limitations should be noted. First, although our data indicate that the CANX P‐domain is required for the detected association between CANX and circFAM65A under our experimental conditions, the underlying molecular mechanism remains unclear. The CANX P‐domain is located within the ER lumen, whereas circFAM65A is predominantly localized in the cytoplasm. Therefore, our findings do not necessarily imply direct RNA recognition by the P‐domain. One possible explanation is that deletion or alteration of the P‐domain affects the conformation or organization of CANX, which may in turn influence its cytoplasmic domain or a cytoplasmic‐facing molecular complex involved in the association with circFAM65A. Further studies are needed to verify these hypotheses. Second, our purified system did not employ detergent‐based membrane protein extraction or membrane reconstitution, and may not fully reproduce the native topology and membrane environment of CANX and FAM134B. Consequently, we cannot rule out the contribution of hydrophobic interactions to the observed in vitro condensation. Future studies incorporating membrane‐mimetic systems will be required to fully elucidate the biophysical nature of these dynamic condensates. Finally, the in vivo efficacy of PLGA/circFAM65A@M‐3E11 was evaluated only in subcutaneous xenograft models. The lack of orthotopic, immunocompetent, or patient‐derived models limits assessment of the native microenvironment and intact immune system, and thus warrants further investigation. In addition, we did not include a corresponding linear RNA control in our therapeutic experiments to definitively confirm that the therapeutic effect is specific to the circular topology. These limitations underscore the need to further clarify the underlying mechanisms and therapeutic potential of circFAM65A.

In conclusion, we identified circFAM65A as a tumor‐suppressive circRNA downregulated in HCC. Mechanistically, circFAM65A promotes CANX condensate formation, and these condensates sequester FAM134B, reducing the FAM134B‐LC3 interaction, which may contribute to the inhibition of ER‐phagy and HCC progression. To translate this finding, we developed a PLGA/circFAM65A@M‑3E11 biomimetic nanosystem for circFAM65A delivery to HCC cells and demonstrated its anti‐tumor efficacy in vivo. Collectively, our findings reveal a mechanism linking circFAM65A‐enhanced CANX condensation to the inhibition of ER‐phagy and HCC progression, and support circFAM65A delivery as a potential therapeutic strategy for HCC.

## 4 Methods

### 4.1 Tissue Samples

In this study, 12 pairs of fresh HCC and normal adjacent tissue samples were collected from Xijing Hospital (Xi'an, China). Informed consent was obtained from all participating patients before the study commenced. The study was approved by the Institutional Ethics Committees of Xijing Hospital and Northwestern Polytechnical University.

### 4.2 Cell Lines and Culture

Human HCC cell lines (Huh7, Hep3B, HepG2, PLC/PRF/5, HCC‐LM3, MHCC‐97L, SK‐Hep1 and SUN368), normal liver cell line (LO2), HEK293T and THP‐1 were obtained from Pricella (Wuhan, China) or Servicebio (Wuhan, China). Culture conditions consisted of DMEM, MEM or RPMI‐1640 with 10% FBS (Vazyme, China) and 1% penicillin/streptomycin (Solarbio, China), in a 5% CO_2_ humidified incubator at 37°C.

### 4.3 Plasmid Construction, Oligonucleotide Synthesis and Transfection

The full‐length sequence of circFAM65A was cloned into the pCDNA‐2.1 vector to achieve overexpression. The expression plasmid of pCD25‐circFAM65A‐MS2bs was synthesized by GENESEED (Guangzhou, China). Expression plasmids for wild‐type CANX (CANX^FL^) and its deletion mutants (CANX^ΔP^, CANX^ΔN^, CANX^ΔTM^, CANX^ΔCyto^, CANX^ΔIDR1^, CANX^ΔIDR2^, and CANX^ΔIDRs^) were constructed by cloning the corresponding sequences into the pCMV‐mNeonGreen‐His, pCMV‐EGFP‐HA, pCMV‐HA, or pCMV‐mCherry vectors. The expression plasmids of pcDNA3.1‐mCherry‐Myc and pCMV‐LC3B‐Flag were obtained from MiaolingBio (Wuhan, China). Small‐interfering RNAs (siRNAs) targeting circFAM65A and CANX were synthesized by General Biol (Anhui, China) (Table S1). Plasmids and oligonucleotides were transfected using Lipofectamine 2000 (Invitrogen, USA) according to the manufacturer's instructions.

### 4.4 Lentivirus Packaging and Infection

Lentivirus particles overexpressing the circFAM65A were synthesized by HanBio (Shanghai, China). Lentiviral vectors pLV3‐puro carrying complementary DNA sequences and pLKO.1‐puro carrying short hairpin RNA (shRNA) sequences (Table S1) were respectively co‐transfected into HEK293T cells with the packaging plasmids pMD2.G and psPAX2. After 48 h, infectious lentivirus particles were collected. Huh7 cells were then infected with the lentivirus in medium containing 6 µg/mL polybrene (Beyotime, China) and selected with 6 µg/mL puromycin (Beyotime, China) for 7 days to establish stable cell lines.

### 4.5 RNA Isolation and Quantitative Real‐Time PCR

Total RNA was isolated using Trizol Reagent (AG, China) and complementary DNA (cDNA) was synthesized with RT reagent kit (AG, China) following the manufacturer's protocol. Quantitative real‐time PCR (qPCR) was conducted using Hieff qPCR SYBR Green Master Mix (YEASEN, China), with GAPDH as the reference gene. Each sample was replicated three times and quantitation data were calculated by the 2^−ΔΔCt^ method. Primer sequences are listed in the Table S1.

### 4.6 RNase R and Actinomycin D Treatment

For RNase R treatment, total RNA was incubated with RNase R (Beyotime, China) at 37°C for 3 h. To determine RNA stability, cells were treated with 2 µg/mL actinomycin D (Sigma, Germany). The digested RNA was then reverse‐transcribed to cDNA and analyzed via RT‐qPCR.

### 4.7 Western Blot

The cells and tissue samples were lysed using RIPA lysis buffer containing 1 mm PMSF (Servicebio, China). The BCA protein assay kit (Beyotime, China) was used for protein quantification. Protein samples were separated by electrophoresis on SDS‐PAGE gels and then transferred onto 0.45 µm PVDF membranes (Millipore, Germany). After blocking with 5% BSA, the membranes were incubated with primary antibodies overnight at 4°C, followed by incubation with HRP‐conjugated secondary antibodies. The signals were visualized using ECL reagents (Vazyme, China). All antibody information is listed in Table S2.

### 4.8 CCK‐8 Assay, EdU Assay, Colony Formation Assay

The growth curves of HCC cells plated in 96‐well plates were determined with the CCK‐8 kit (AG, China). For the colony formation assay, transfected HCC cells were seeded into 6‐well plates (1,000 cells/well) and cultured for two weeks. The colonies were subsequently fixed in 4% paraformaldehyde and visualized by staining with 0.5% crystal violet (Beyotime, China). Cell proliferation was further assessed using the EdU assay kit (Beyotime, China) following the manufacturer's protocol and images were visualized by fluorescence microscopy (Olympus, Japan). All experiments were performed at least in triplicate.

### 4.9 Transwell Assay and Wound Healing Assay

For the migration assay, transfected HCC cells (50 000 cells/well) were suspended in 200 µL of serum‐free medium and added to the upper chamber of 24‐well transwell inserts (8 µm pore size; Corning, USA). The lower chamber was filled with 600 µL of complete medium. After 48 h of incubation, cells remaining on the upper surface were removed by scrubbing, whereas cells that had migrated to the lower surface were fixed with 4% paraformaldehyde and stained with 0.5% crystal violet (Beyotime, China). In wound healing assays, transfected cells were seeded in 6‐well plates and cultured to full confluence. The cells were scratched and then maintained in serum‐free medium for 48 h. Images captured at 0 h and 48 h were used to assess wound width changes. All experiments were performed at least in triplicate.

### 4.10 Flow Cytometry Analysis (FACS) of Apoptosis

Apoptotic cells were detected using Annexin V‐APC/PI Apoptosis Detection Kit (Vazyme, China) according to the manufacturer's instructions. Briefly, cells were collected with EDTA‐free trypsin, washed with PBS, and resuspended in binding buffer. After staining with Annexin V‐APC and PI for 15 min in the dark, apoptosis was analyzed by Beckman CytoFLEX platform. All experiments were performed at least in triplicate.

### 4.11 Hypro‐MS Analysis

Hypro‐MS analysis was performed by Aksomics (Shanghai, China). Briefly, DIG‐labeled probes were designed based on the circFAM65A sequence. After cell fixation and permeabilization, the probes were incubated with the cells. Hypro enzyme and reaction substrates (biotin‐phenol, H_2_O_2_, and biotin) were then added to carry out the proximity labeling reaction. Finally, the proteins were isolated and subjected to mass spectrometry analysis.

### 4.12 Turbo‐ID Proximity Labeling Assay

Turbo‐ID proximity labeling assay was performed as previously described [51]. Briefly, cells stably expressing TurboID‐MS2 were cultured in 100 µm biotin at 37°C for 10 min. Then, cells were chilled on ice 15–30 s, washed with ice‐cold PBS, and lysed in RIPA buffer containing 1 mm PMSF and protease inhibitor. After incubation with streptavidin beads at 4°C for 2 h, the beads were washed, and biotinylated proteins were eluted for western blotting.

### 4.13 Fluorescence In Situ Hybridization (FISH)

RNA FISH was conducted by Servicebio (Wuhan, China) using a specific probe for the back‐splice junction of circFAM65A. In brief, cells plated on coverslips in 6‐well plates were fixed with in situ hybridization fixative, permeabilized with 0.1% Triton X‐100, and hybridized with the probe at 37°C overnight. Nuclei were stained with DAPI, and images were acquired by confocal microscopy (OLYMPUS, Japan).

### 4.14 Immunofluorescence (IF)

Cells cultured on sterile glass coverslips were fixed with 4% paraformaldehyde for 30 min at room temperature, permeabilized with 0.3% Triton X‐100 for 15 min, and blocked with 1% BSA for 1 h. Then, cells were incubated overnight at 4°C with the primary antibody, followed by Alexa Fluor‐labeled secondary antibody for 1 h at room temperature in the dark. Nuclei were stained with DAPI, and images were acquired by confocal microscopy (OLYMPUS, Japan).

### 4.15 RNA Pull‐Down

RNA pull‐down assays were performed using the RNA‐Protein Pull Down Kit (Sangon Biotech, China) according to the manufacturer's instructions. Briefly, 3′‐biotin‐labeled probes targeting circFAM65A were incubated with streptavidin‐coated magnetic beads. Cell lysates were then incubated overnight with the bead–probe complexes. After washing, the bound proteins were eluted and analyzed by Western blotting.

### 4.16 RNA Immunoprecipitation Assay (RIP)

The RIP assay was performed using the RIP‐Assay Kit (MBL, Japan) according to the manufacturer's instructions. Briefly, protein A/G magnetic beads were incubated with specific antibodies or IgG, and then incubated overnight with HCC cell lysates. After washing, RNA was extracted from the immunoprecipitated complexes and analyzed by qRT‐PCR.

### 4.17 RNA Sequencing

Total RNA was isolated from control Huh7 cells and those overexpressing circFAM65A using Trizol reagent. Libraries were constructed and sequencing was performed by NovelBio (Shanghai, China). Pathway enrichment analysis was carried out using the clusterProfiler package.

### 4.18 Autophagy Assay

Huh7 cells stably expressing RFP‐GFP‐LC3, RFP‐GFP‐KDEL, or mCherry‐Sec61B were transfected with the corresponding plasmids. After 48 h, the medium was changed to EBSS to induce autophagy. The fluorescence of RFP‐GFP‐LC3 and RFP‐GFP‐KDEL puncta was observed using confocal microscopy (OLYMPUS, Japan). The number of RFP^+^GFP^−^ puncta were counted in 20 independent cells per experiment. For mCherry‐Sec61B assay, free mCherry was detected by western blotting.

### 4.19 Co‐Immunoprecipitation Assay (Co‐IP)

Transfected cells were lysed in IP lysis buffer with PMSF (Beyotime, China) on ice for 20 min. After centrifugation at 12 000 rpm for 20 min at 4°C, supernatants were collected and incubated with specific antibodies or IgG for 4 h at 4°C, then with protein A/G beads (Thermo Fisher, USA) overnight. After washing, the immunoprecipitated proteins were eluted in SDS‐PAGE sample loading buffer (Beyotime, China), boiled at 100 °C for 10 min, and detected by western blot.

### 4.20 Protein Purification and In Vitro Phase Separation Assay

Recombinant CANX proteins, their mutants, and FAM134B proteins were cloned into pET‐28a plasmids and expressed in E. coli BL21. Bacterial cultures were grown to an OD600 of 0.6–0.8 at 37°C, then induced with 0.1 mm IPTG (Beyotime, China) and further incubated at 16°C for 16 h for protein expression. Bacteria were harvested and resuspended in the lysis buffer (50 mm Tris‐HCl pH 8.0, 500 mm NaCl, and 20 mm imidazole pH 8.0) and then lysed by sonication followed by centrifugation at 18 000 rpm for 20 min at 4°C. The supernatant was purified using HisTrap HP column (Cytiva, USA) and eluted using elution buffer (50 mm Tris‐HCl pH 8.0, 500 mm NaCl, and 300 mm imidazole pH 8.0). Notably, no detergents, liposomes, or nanodiscs were included during the solubilization and purification processes.

Purified proteins were diluted at the indicated concentrations with prechilled PBS. Polyethylene glycol (PEG8000) was supplemented to a final concentration of 10%. The mixture was then loaded into a flow chamber constructed by attaching double‑sided adhesive tape onto a glass slide. The images were immediately acquired by Laser Scanning Confocal Microscope FV3000 (OLYMPUS, Japan).

### 4.21 Fluorescence Recovery After Photobleaching (FRAP)

FRAP assay was performed using a laser scanning confocal microscope FV3000 (OLYMPUS, Japan). For cellular FRAP, cells were seeded into a confocal dish (Corning). An appropriate region of interest was photobleached using a 488 nm laser at 20% power for 1 s. For in vitro FRAP assays, droplets were photobleached with 70% laser power for 1 s using a 488 nm laser. Time‑series images were acquired every 5 s after bleaching.

### 4.22 Production and Purification of circRNA

The in vitro transcription of circFAM65A was performed and purified by Yaohabio (Jiangsu, China). UV spectrophotometry was used to assess purity from protein contaminants, yielding an A260/A280 ratio of 2.277. The integrity and size of RNA samples were further validated by agarose gel electrophoresis. Sanger sequencing confirmed the expected circFAM65A back‐splice junction sequence.

### 4.23 Cell Membrane Derivation

THP‐1 cells stably expressing the 3E11 antibody gene (1 × 10^7^ cells) were washed three times with PBS and then resuspended in 1 mL of hypotonic lysis buffer (20 mm Tris‐HCl, pH 7.5, 10 mm KCl, 2 mm MgCl_2_). The cells were ultrasonically fragmented, followed by centrifugation at 3,500 g for 5 min at 4°C. The supernatant was subsequently centrifuged at 16 900 g for 30 min at 4°C, and the pellets were washed with DNase/RNase‐free water. Finally, the pellet was stored at −80°C for subsequent analysis.

### 4.24 PLGA/circFAM65A@M‐3E11 Preparation

CircFAM65A in PVA‐containing RNase‐free water and PLGA in acetone were combined at a circFAM65A: PLGA mass ratio of 1:10, ultrasonicated, and stirred in the dark at room temperature for 4 h to yield PLGA/circFAM65A nanoparticles. Nanoparticles were collected by centrifugation at 12 000 rpm for 10 min at 4°C. The PLGA/circFAM65A nanoparticles were then mixed with extracted M‐3E11 at a mass ratio of 1:5 in PBS, ultrasonicated at low temperature for 2 min, and extruded through 400 nm membranes with 10 cycles to promote membrane fusion.

### 4.25 Targeting Validation

Nanoparticles loaded with DiO dye (Beyotime, China) were prepared. Cell membranes were stained with DiI dye (Thermo Fisher, USA). The nanoparticles and stained cells were then co‑incubated for 30 min, followed by washing and fixation for microscopic observation to examine nanoparticle binding to the cell membrane. Similarly, lysosomes were labeled using a LysoTracker kit (Beyotime, China). In this case, nanoparticles were co‑incubated with cells for 2 h before microscopic observation to detect nanoparticle entry into cells. Targeting efficiency was also assessed by flow cytometry.

For in vivo experiments, nanoparticles loaded with ICG dye (MCE, USA) were prepared. HCC subcutaneous xenograft mice were administered either PLGA/ICG@M‐3E11 or PLGA/ICG@M (5 mg/kg PLGA) via the tail vein. The distribution and metabolism of the nanoparticles in vivo were monitored using a small animal in vivo imaging system (IVIS Spectrum) at indicated time points.

### 4.26 Immunohistochemistry (IHC)

Tissue specimens were fixed with formalin, dehydrated through a graded ethanol series, and embedded in paraffin. Sections were then incubated overnight at 4°C with primary antibodies targeting CANX, Ki‐67, cleaved caspase3, LC3B, and P62, followed by incubation with secondary antibodies at room temperature. Subsequently, DAB and hematoxylin staining were performed, and images were captured under a microscope.

### 4.27 Animal Study

All animal procedures were approved by the Laboratory Animal Welfare and Ethics Committee of Northwestern Polytechnical University. Male BALB/c nude mice (6 weeks old) were obtained from CAVENS (Changzhou, China). Mice were randomly assigned to treatment groups. To establish a subcutaneous xenograft model, Huh7 cells (1 × 10^7^) were resuspended in medium containing 25% Matrigel (Vazyme, China) and injected subcutaneously into the axillary region of the mice. For in vivo treatment, PLGA/circFAM65A@M‑3E11 or PLGA@M‑3E11 (5 mg/kg PLGA) was administered via tail vein injection every 8 days for three times after tumor inoculation. Tumor volumes were measured on days 8, 16, 24, and 32. Mice were euthanized on day 32 after inoculation, and subcutaneous tumors were harvested for further analysis.

### 4.28 Organ Toxicity Evaluation

For organ toxicity evaluation, healthy BALB/c nude mice received weekly tail vein injections of PBS or PLGA/circFAM65A@M‐3E11 for three consecutive weeks. After euthanasia, serum was collected by centrifugation (3000 rpm, 10 min, 4°C) and used to measure alanine aminotransferase (ALT), aspartate aminotransferase (AST), and renal function markers UREA, creatinine (CREA) with commercial kits on a multifunctional biochemical analyzer. Major organs (heart, liver, spleen, lungs, kidneys, and brain) were harvested for H&E staining and histopathological evaluation.

### 4.29 Statistical Analysis and Quantification

Statistical analysis was performed using GraphPad Prism 8.0. For comparisons between two independent groups, two‐tailed unpaired Student's t‐tests were used. For paired or matched samples, two‐tailed paired Student's t‐tests were used. For comparisons among three or more groups, one‐way ANOVA followed by Tukey's or Dunnett's multiple comparisons test was used. For longitudinal or repeated‐measures data, two‐way repeated‐measures ANOVA followed by Sidak's multiple comparisons test was used.

Quantitative data are presented as mean ± s.d. from three independent experiments. For qRT‐PCR, CCK‐8, and flow cytometry assays, three technical replicates were included within each independent biological experiment. For imaging‐based assays, quantification was performed by analyzing multiple random fields per sample. For in vivo experiments, each mouse was considered an independent biological replicate, and all animals received the same treatment schedule and experimental procedures. The detailed sample sizes, independent biological replicates, and statistical tests used are specified in the corresponding figure legends.

ImageJ software was used for quantification of EdU assay, colony formation assay, transwell assay, wound healing assay, puncta number. Identical analysis parameters were applied across all samples to maintain consistency and comparability.

## Author Contributions

R.W., W.W., Q.W., and Y.F. conceived and designed the study; R.W. performed the study, analyzed the data, and wrote the manuscript; G.L. and L.Y. assisted in conducting experiments and performed animal experiments; G.Y., H.Q., and Y.S. analyzed and interpreted the data; D.S., N.F., N.J., L.L., and L.Z. reviewed and revised the manuscript; Y.S. and W.H. assisted in conducting experiments; W.W., Q.W., and Y.F. provided funding support; W.W. supervised the study. All authors reviewed and approved the final manuscript.

## Funding

This study was supported by the National Natural Science Foundation of China (82573816, 82220108004, 82173204, 82606380), the Shaanxi Provincial Science and Technology Department (Innovation Capability Support Program, 2023‐CX‐TD‐72; Innovation Capability Enhancement Program, 2024TD‐08; Natural Science Basic Research Program, 2025JC‐YBQN‐1160), and the China Postdoctoral Science Foundation (Postdoctoral Fellowship Program of CPSF, GZC20233519).

## Ethics Statement

The use of clinical samples was approved by the Institutional Ethics Committee of the Xijing Hospital and Northwestern Polytechnical University, and informed consent was obtained from all patients. The experiments involving animals were approved by the Laboratory Animal Welfare and Ethics Committee of the Northwestern Polytechnical University.

## Conflicts of Interest

The authors declare no conflicts of interest.

## Supporting information




**Supporting File**: advs78469‐sup‐0001‐SuppMat.doc.

## Data Availability

The authors declare that data supporting the findings of this study are available within the article and its supplementary information files.
