# Supplementary material for: CircFAM65A/CANX Condensates Sequester FAM134B to Inhibit ER‐phagy and Suppress Hepatocellular Carcinoma Progression
Source: Adv Sci (Weinh). 2026 Oct 27:e78469. Online ahead of print. doi: 10.1002/advs.78469 (PMC13712397; doi:10.1002/advs.78469)
Supplement: Supplementary file 1 [file ADVS-9999-e78469-s001.doc]

**Supporting Information for**

**CircFAM65A/CANX condensates sequester FAM134B to inhibit ER-phagy and suppress hepatocellular carcinoma progression**

Wenxiu Ru^#^, Lunbiao Gan^#^, Yu Lu^#^, Yujing Guo, Qian Huang, Sha Yin, Fengze Nie, Sijia Di, Jiaqi Ning, Siyu Yao, Huanhuan Wan, Le Liu, Zehua Liu, Fa Yang, Weijun Qin, Weihong Wen

Supplementary figures……………………………………………….…….......…....2

Supplementary tables………………………………...……...........………….……..14

Uncropped Gels and Blots images.............................................................................15

**Supplementary figure**

**Figure S1. Characterization of circFAM65A in HCC, related to Figure 1.**

**a** RT-PCR analysis of circFAM65A and FAM65A mRNA using divergent and convergent primers. **b** Analysis of circFAM65A expression in HCC cell lines using qRT-PCR. **c, d** Analysis the stability of circFAM65A and FAM65A after treatment with RNase R (c) and actinomycin D (d). **e, f** Schematic illustration of the MS2-GFP live-cell imaging system targeting circRNA (e) and representative images of circFAM65A subcellular localization (f). Scale bar, 5 μm. All microscopy images are representative of n = 3 independent experiments. Data are mean ± s.d. from three independent experiments. Statistical analysis was performed using one-way ANOVA followed by Dunnett's multiple comparisons test (b), unpaired two-tailed Student’s t-test (c), or two-way ANOVA followed by Sidak’s multiple comparisons (d).**P* < 0.05 and ****P* < 0.001.

**Figure S2. circFAM65A inhibits cell proliferation and migration in HCC, related to Figure 1.**

**a, b** Efficiency of circFAM65A overexpression in HepG2 and HCC-LM3 cells (a) and of circFAM65A knockdown in Huh7 and Hep3B cells (b). **c-e** CCK-8 assays (c), colony formation assays (d) and EdU assays (e) in circFAM65A-overexpressing cells. **f-h** CCK-8 assays (f), colony formation assays (g) and EdU assays (h) in circFAM65A-knockdown cells. **i, j** Transwell migration assays in circFAM65A-overexpressing cells (i) and in circFAM65A-knockdown cells (j). **k, l** Wound healing assays in circFAM65A-overexpressing cells (k) and in circFAM65A-knockdown cells (l). Data are mean ± s.d. from three independent experiments. Statistical analysis was performed using one-way ANOVA followed by Dunnett's multiple comparisons test (a, b, g, h, j), unpaired two-tailed Student’s t-test (d, e, i, k), or two-way ANOVA followed by Šídák's multiple comparisons (c, f). **P*< 0.05, ***P* < 0.01 and ****P* < 0.001.

**Figure S3. Hypro‑MS profiling and the IDR features of CANX, related to Figure 2.**

**a** Venn diagram showing the number of proteins interacting with circFAM65A. **b** GO enrichment analysis of circFAM65A-interacting proteins. **c** Sequence features of human CANX predicted by PONDR. **d** Three-dimensional structure of CANX predicted by MobiDB. IDR highlighted in red. **e** Schematic representation of the circRNA-TurboID proximity labeling system. **f** RIP assay showing the association between circFAM65A and CANX in Huh7 cells. **g** Live-cell confocal imaging of circFAM65A (MS2-GFP system, green) co-localization with different CANX-mCherry truncates (red). Scale bar, 5 μm. All microscopy images are representative of n = 3 independent experiments. Data are mean ± s.d. from three independent experiments. Statistical analysis was performed using unpaired two-tailed Student’s t-test (f). ****P* < 0.001.

**Figure S4. CANX increases cell proliferation, migration while suppressing cell apoptosis in HCC, related to Figure 3.**

**a** ROC analysis for the prediction performance of the CANX from TCGA data. **b** Survival analysis of HCC patients based on CANX expression levels from TCGA data. **c** IHC anlysis of CANX using the Human Protein Atlas Database. **d** Expression of CANX protein in HCC cell lines. **e, f** Efficiency of CANX knockdown in Huh7 cells (e) or overexpression in Hep3B cells (f). **g, h** CCK-8 assays (g) and colony formation assays (h) in cells overexpressing or knocking down CANX. **i, j** Transwell migration assays (i) and wound healing assays (j) in cells overexpressing or knocking down CANX. **k** Flow cytometry analysis of apoptosis in cells overexpressing or knocking down CANX. Data are mean ± s.d. from three independent experiments. Statistical analysis was performed using two-way ANOVA followed by Šídák's multiple comparisons (g), one-way ANOVA followed by Dunnett's multiple comparisons test or unpaired two-tailed Student’s t-test (h-k). **P* < 0.05, ***P* < 0.01 and ****P* < 0.001.

**Figure S5. Autophagy is associated with a poor prognosis in HCC, related to Figure 3.**

**a** Prognostic model for HCC patients based on autophagy-related genes using TCGA data. **b** Representative IHC staining of LC3B and p62 in normal samples and HCC tissues. Scale bar, 50 μm. **c** Correlation analysis between CANX expression and autophagy from TCGA data. **d** Display of the correlation between CANX and autophagy-related genes. **e** Western blot analysis of LC3-II expression changes in Huh7 cells following CANX overexpression or knockdown, under normal conditions or treatment with the autophagy inhibitor CQ (10 μM). All microscopy images and blots are representative of n = 3 independent experiments.

**Figure S6. CANX condensation is dependent on its IDR2 domain, and circFAM65A promotes its condensate formation, related to Figure 4.**

**a** Representative fluorescence imaging of CANX staining by IF in HCC cells. **b** Coomassie blue staining image of purified CANX-EGFP protein and its mutant constructs. **c** Brightfield microscopy images showing purified CANX droplets fusing into larger ones over time in vitro. **d** Droplet formation of purified CANX or CANX-EGFP in vitro. **e** Schematic diagrams of CANX and indicated mutants. **f** Representative fluorescence images showing phase separation of CANX-mNeonGreen and its mutant constructs in HCC cells. Scale bar, 10 μm. **g** Droplet formation of purified CANX-EGFP and its mutant constructs in vitro. Scale bar, 10 μm. **h, i** Analysis of CANX expression in circFAM65A-overexpressing HCC cells by RT-qPCR (h) and western blotting (i). **j** Droplet formation of purified CANX-EGFP with 50 ng/μL circFAM65A, with or without RNase A or RNase R, in vitro. Scale bar, 5 μm. **k** Representative fluorescence images showing CANX-mNeonGreen puncta in circFAM65A-overexpressing Huh7 cells or cells treated with 1 μM TG. **l** Representative fluorescence images showing CANX-mNeonGreen puncta in circFAM65A-overexpressing Huh7 cells treated with 5% 1,6-HD or 0.01% Tween-20. Scale bar, 5 μm. **m** Representative fluorescence imaging of p62 staining and CANX-mNeonGreen puncta in Huh7 cells with or without circFAM65A overexpression. Scale bar, 5 μm. Statistical analysis was performed using two-tailed Student’s t-test (h). All microscopy images and blots are representative of n = 3 independent experiments.

**Figure S7. circFAM65A promotes cell apoptosis and inhibits ER-phagy in HCC cells, related to Figure 5.**

**a** Functional enrichment analysis of differentially expressed genes in circFAM65A-overexpressing Huh7 cells compared to control. **b** Western blot analysis of LC3-II and p62 expression changes following circFAM65A overexpression or knockdown under EBSS-treated conditions. **c** Western blot analysis of LC3-II expression changes in Huh7 cells following circFAM65A overexpression or knockdown, under normal conditions or treatment with the autophagy inhibitor CQ (10 μM). **d** Representative images of the autophagic flux using the RFP-GFP-LC3 reporter in circFAM65A‑overexpressing Huh7 cells under EBSS-treated conditions. Scale bar, 10 μm. Quantification of RFP⁺GFP^-^ puncta per cell is shown in the right panel. **e** Western blot analysis of free mCherry levels using the mCherry-Sec61B reporter in circFAM65A-overexpressing and circFAM65A‑knockdown Huh7 cells under EBSS-treated conditions. **f** Representative images of the autophagic flux using the RFP-GFP-KDEL reporter in circFAM65A-overexpressing Huh7 cells under EBSS-treated conditions. Scale bar, 10 μm. Quantification of RFP⁺GFP^-^ puncta per cell is shown in the right panel. **g** Flow cytometry analysis of apoptosis in Huh7 cells overexpressing or knocking down circFAM65A. **h** IHC staining of LC3B and p62 in subcutaneous tumors from circFAM65A-overexpressing xenografts. Scale bar, 50 μm.  All microscopy images and blots are representative of n = 3 independent experiments. Data are mean ± s.d. from three independent experiments. Statistical analysis was performed using unpaired two-tailed Student’s t-test (d, f, g) or one-way ANOVA followed by Dunnett's multiple comparisons test (g). ***P* < 0.01 and ****P* < 0.001.

**Figure S8. circFAM65A counteracts CANX-induced proliferation and migration and restores apoptosis in HCC cells, related to Figure 5.**

**a**-**c** CCK-8 assays (a), transwell migration assays (b), and flow cytometry analysis of apoptosis (c) in CANX-knockout Huh7 cells with CANX overexpression alone, or co-expression of circFAM65A with CANX^FL^, CANX^ΔP^ or CANX^ΔIDR2^. **d** Western blot detection of ATF4 and CHOP expression in TG-treated (1 μM) Huh7 cells after overexpression of CANX^FL^ alone, or co-transfection of circFAM65A with CANX^FL^, CANX^ΔP^ or CANX^ΔIDR2^. Data are mean ± s.d. from three independent experiments. Statistical analysis was performed using two-way ANOVA followed by Šídák's multiple comparisons (a), or one-way ANOVA followed by Tukey’s multiple comparisons test (c). ****P* < 0.001.

**Figure S9. CANX interacts with FAM134B in HCC cells, related to Figure 6.**

**a** Western blotting of HA-IP assay showing the interactions between CANX and ER-phagy receptor in CANX-overexpressing Huh7 cells. **b** Co-IP assay to detect the interaction between CANX-HA and FAM134B-Myc in HEK293T cells. **c** Co-IP analysis of CANX and FAM134B in Huh7 cells. **d** HA-IP assay followed by western blotting to detect the interaction between CANX‑HA and FAM134B-Myc in HEK293T cells with or without circFAM65A. **e** Coomassie blue staining image of purified FAM134B-mCherry protein. **f** Western blotting of HA-IP assay showing the interactions between CANX-HA and FAM134B-Myc in HEK293T cells co-expressing circFAM65A, with or without EBSS treatment. All blots are representative of n = 3 independent experiments.

**Figure S10. Characterization and safety analysis of PLGA/circFAM65A@M-3E11, related to Figure 7.**

**a, b** Size distributions (a) and zeta potentials (b) of PLGA and PLGA@M-3E11 nanoparticles. **c** Representative fluorescence imaging showing colocalization of PLGA@M-3E11 nanoparticles with lysosomes. Scale bars, 10 μm. **d** Detection of in vitro synthesized circFAM65A by gel electrophoresis. **e** Back-splice junction site of exogenous circFAM65A synthesized in vitro was verified by Sanger sequencing. **f** Schematic illustration of the PLGA/circFAM65A@M-3E11 treatment. **g** FISH analysis of circFAM65A expression in subcutaneous tumor. Scale bar, 50 μm. **h** IHC staining of KI67, Cleaved Caspase3 in subcutaneous tumor. Scale bar, 50 μm. **i** Blood biochemistry measurements, including hepatic function markers ALT, AST, and renal function markers UREA, CREA, were performed in healthy BALB/c nude mice following treatment. **j** Hematoxylin and eosin (H&E) staining of major organs harvested following treatment. All microscopy images and blots are representative of n = 3 independent experiments. Data are mean ± s.d. from three independent experiments. Statistical analysis was performed using unpaired two-tailed Student’s t-test (i).

**Supplementary tables**

**Table S1. Primer and oligo sequences**

| Name | Sequence |
| --- | --- |
| GAPDH-qPCR-F | CATCTTCTTTTGCGTCGCCA |
| GAPDH-qPCR-R | TTAAAAGCAGCCCTGGTGACC |
| CircFAM65A-qPCR-F | TGGTTGTGGTTTAGGAGGGGG |
| CircFAM65A-qPCR-R | ACCTCACCTGCCTTTTACCTGA |
| FAM65A-qPCR-F | CCAGGAGTTTCCCGGTCTTC |
| FAM65A-qPCR-R | CTTCCAAGTAGGCCGTCAGG |
| Si-circFAM65A-1 | GGCAGGTGAATAAATGGGT |
| Si-circFAM65A-2 | GCAGGTGAATAAATGGGTG |
| CircFAM65A probe | CATTTATTCACCTGCCTTTGGAATG |
| Si-CANX-1 | CCAAGCCUCUCAUUGUUCATT |
| Si-CANX-2 | GAGGACUAUAAACUGCACUTT |
| shCANX | CCAAGCCUCUCAUUGUUCATT |
| Negative control | UUCUCCGAACGUGUCACGUTT |

**Table S2. List of antibodies in this study**

| Name | Supplier | Cat No. |
| --- | --- | --- |
| CANX | Proteintech | 10427-2-AP |
| HA-Tag | ABclonal | AE105 |
| Flag-Tag | HUABIO | HA724169 |
| Myc-Tag | ABclonal | AE070 |
| LC3 | Proteintech | 14600-1-AP |
| p62 | HUABIO | HA721171 |
| mCherry | Epizyme Bio | LF312S |
| CLIMP63 | ZENBIO | 161564 |
| Ki67 | Abcam | ab15580 |
| Cleaved Caspase3 | Proteintech | 25128-1-AP |
| FAM134B | Proteintech | 21537-1-AP |
| SEC62 | ZENBIO | R30231 |
| RTN3L | HUABIO | HA721342 |
| CCPG1 | Proteintech | 13861-1-AP |
| GAPDH | Proteintech | 60004-1-Ig |
| IgG | Proteintech | 30000-0-AP |
| CHOP | Proteintech | 15204-1-AP |
| ATF4 | Proteintech | 10835-1-AP |
| [Anti-Rabbit IgG Light Chain](https://abclonal.com.cn/catalog/AS126) | ABclonal | AS126 |
| Goat anti-Rabbit IgG | HUABIO | HA1001 |
| Goat anti-Mouse IgG | HUABIO | HA1006 |

**Uncropped Gels and Blots images**

**Figure 2d**

100

70

kDa

**Figure 2g**

IP-HA

200

100

70

kDa

Input-HA	Input-GAPDH

200

100

70

kDa

40

kDa

**Figure 3b**

CANX

100

kDa

GAPDH

**Figure 3h**

Hep3B-LC3	Huh7-LC3

15

kDa

kDa

15

Hep3B-p62	Huh7-p62

kDa

kDa

70

70

Hep3B-CANX	Huh7-CANX

70

kDa

70

kDa

Hep3B-GAPDH	Huh7-GAPDH

40

kDa

kDa

40

**Figure 3l**

SiCANX: mCherry-long	mCherry-short

40

25

kDa

kDa

40

25

OeCANX: mCherry-long	mCherry-short

kDa

40

25

40

25

kDa

70

siCANX-CLIMP63	oeCANX-CLIMP63

70

kDa

kDa

70

siCANX-GAPDH	oeCANX-GAPDH

kDa

kDa

40

40

**Figure 5a**

LC3	p62

70

15

kDa

kDa

HA	GAPDH

40

100

70

kDa

kDa

**Figure 5d**

mCherry-long	mCherry-short

40

25

kDa

40

25

kDa

CLIMP63	GAPDH

70

kDa

kDa

40

**Figure 6e**

IP:HA IB:Myc	IP:HA IB:HA

kDa

kDa

70

70

Input: HA	Input: Myc

kDa

kDa

70

70

**Figure 6h**

kDa

mCherry-long	mCherry-short

40

25

kDa

40

25

kDa

CLIMP63	HA

70

kDa

70

kDa

100

GAPDH

40

kDa

**Figure 6i**

mCherry-long	mCherry-short

40

kDa

40

25

kDa

25

CLIMP63	HA

kDa

70

kDa

100

GAPDH

kDa

40

**Figure 6j**

IP: Myc IB: Flag	IP: Myc IB: Myc

70

kDa

25

kDa

Input: Flag	Input: Myc

25

kDa

kDa

40

Input:HA	Input: GAPDH

100

kDa

kDa

40
